# Clinical efficacy and safety of Chinese herbal injections in combination with platinum-based chemotherapy for advanced non-small cell lung cancer: a systematic review and meta-analysis of 140 randomized controlled trials

**DOI:** 10.3389/fonc.2024.1307836

**Published:** 2024-02-02

**Authors:** Kangdi Cao, Shuaihang Hu, Dandan Wang, Chenxi Qiao, Zhuo Wang, Jinkun Wang, Wei Hou

**Affiliations:** ^1^ Department of Oncology, Guang’anmen Hospital, China Academy of Chinese Medical Sciences, Beijing, China; ^2^ Department of Graduate School, Beijing University of Chinese Medicine, Beijing, China

**Keywords:** advanced non-small cell lung cancer, Chinese herbal injection, meta-analysis, platinum-based chemotherapy, randomized controlled trial, systematic review

## Abstract

**Background and aim:**

Chinese herbal injection (CHI) is a widely used preparation for advanced non-small cell lung cancer (NSCLC) treatment to alleviate the adverse drug reactions and enhance the clinical efficacy of chemotherapy. However, its efficacy and safety in combination with platinum-based chemotherapy (PBC) remain poorly understood owing to the lack of high-level evidence in the face of a wide variety of CHIs. Therefore, in this study, we aimed to explore the efficacy and safety of CHIs in combination with PBC regimens in the treatment of mid- and advanced NSCLC.

**Methods:**

Systematic evaluation and meta-analysis were conducted as per the Preferred Reporting Project for Systematic Evaluation and Meta-Analysis Protocols (PRISMA-P). Seven databases were comprehensively searched for relevant randomized controlled trials (RCTs) through August 1, 2022. The quality of each study was evaluated based on the Cochrane Handbook for Systematic Reviews of Interventions. Statistical analysis was performed using Revman 5.3, with dichotomies expressed as risk ratio (RR) and 95% confidence interval (CI). Objective response rate (ORR) and disease control rate (DCR) were selected as the primary outcomes, with quality of life (QoL) and toxic side effects as secondary outcomes.

**Results:**

A total of 140 RCTs were included in this study. The results of the meta-analysis suggested that, compared with PBC alone, PBC combined with CHIs significantly improved the ORR (RR=1.35, 95% CI: 1.30–1.41, P<0.001), DCR (RR=1.15, 95% CI: 1.13–1.18, P<0.001) and QoL (RR=1.29, 95% CI: 1.24–1.33, P<0.001). Moreover, the combination treatment reduced chemotherapy-induced leukopenia (RR=0.69, 95% CI: 0.64–0.75, P<0.001), anemia (RR=0.70, 95% CI: 0.62–0.79, P<0.001), thrombocytopenia (RR=0.68, 95% CI: 0.62–0.75, P<0.001), nausea and vomiting (RR=0.69, 95% CI: 0.63–0.76, P<0.001), diarrhea (RR=0.59, 95% CI: 0.48–0.73, P<0.001), and constipation (RR=0.68, 95% CI: 0.54–0.86, P=0.001).

**Conclusion:**

According to the available evidence, CHIs in combination with PBC can improve clinical efficacy and reduce the toxic side effects in the treatment of advanced NSCLC. However, considering the study’s limitations, more rigorous and high-quality studies are needed to further confirm the results.

**Systematic review registration:**

https://inplasy.com/inplasy-2022-1-0104/, identifier INPLASY202210104.

## 1 Introduction

The 2020 Global Cancer Report showed 2.1 million new cases and 1.8 million deaths from lung cancer in 2018. Lung cancer remains the leading cause of cancer deaths, accounting for approximately one-fifth of all cancer deaths (1). Non-small cell lung cancer (NSCLC) accounts for approximately 85% of lung cancers. Most patients with NSCLC are at an advanced stage at the time of discovery, losing optimal timing for surgical treatment (2). Platinum-based chemotherapy (PBC) remains the preferred treatment for patients with advanced NSCLC who are not driven by a positive gene. PBC is based on cisplatin or carboplatin and is often used in combination with pemetrexed, docetaxel, gemcitabine, paclitaxel, and vincristine. Previous clinical studies found that the median progression-free survival (PFS) of pemetrexed plus cisplatin or carboplatin in the treatment of advanced NSCLC was 6 months and 4.7 months, respectively, and the 1-year survival rates were 45.7% and 39.2% (3), respectively. The median survival time (overall survival, OS) of gemcitabine plus cisplatin or carboplatin in the treatment of advanced NSCLC was 8.2 months (4), which has been proven clinically effective. Therefore, the efficacy of PBC in treating middle and advanced NSCLC is considerable; moreover, OS and health-related quality of life (QoL) do not exhibit differences between carboplatin- and cisplatin-based chemotherapy (5, 6). However, although PBC kills cancer cells, it also damages the human body, causing toxic side effects such as bone marrow suppression, nausea and vomiting, and liver and kidney toxicity, adversely affecting the smooth chemotherapy cycle and severely reducing the QoL of patients (7). Therefore, searching for effective NSCLC complementary alternative therapies remains one of the research hotspots.

Traditional Chinese medicine (TCM) has long been used as a supplement and alternative therapy in NSCLC treatment. Previous studies have shown that TCM can inhibit tumor growth, modulate immune function, increase sensitivity to chemotherapy when combined with it, reduce toxic side effects, and improve the QoL of patients (8, 9). Chinese herbal injection (CHI) is the combination of modern pharmaceutical technology and traditional Chinese prescription, where a sterile preparation is extracted from Chinese herbal medicine. It has the advantages of convenient application, immediate effect, and clear indication and has been widely used in clinical practice. Moreover, TCM has proved to be effective in treating advanced NSCLC. Various CHIs have been developed based on TCMs such as ginseng, *Sophora flavescens*, coix seed, and toad cake. Such CHIs include Compound Kushen injection, Aidi injection, Kanglaite injection, and Shenqifuzheng injection. Previous studies have shown that these CHIs when combined with PBC can positively increase efficacy and reduce chemotherapy-associated toxicity (10–12).

**Figure 1:**
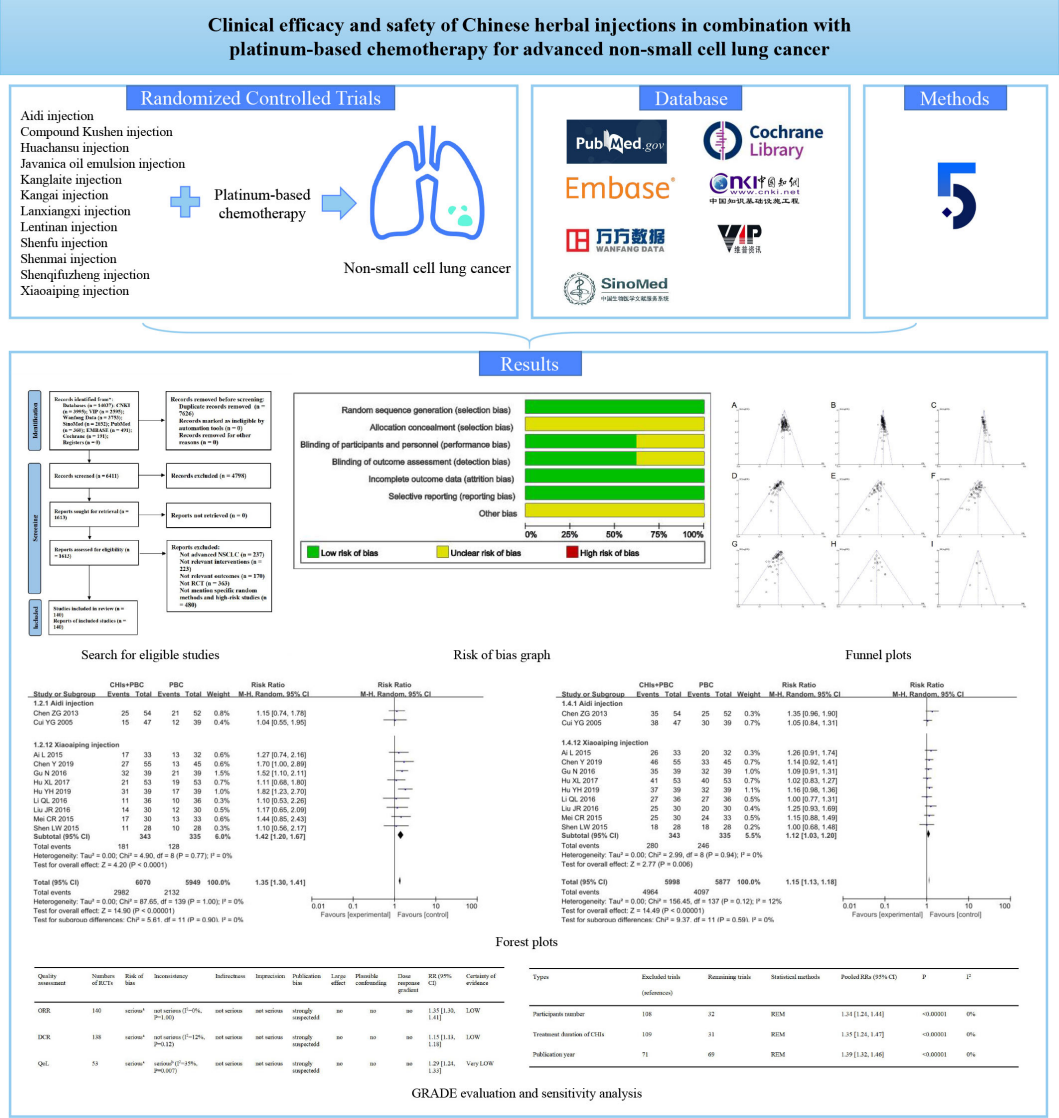
Graphical abstract of the meta-analysis. Meta-analytic process of clinical efficacy and safety of Chinese herbal injections in combination with platinum-based chemotherapy for advanced non-small cell lung cancer.

However, despite the wide clinical application of CHIs, their efficacy and safety in combination with PBC need to be further evaluated given the lack of high-level evidence or clinical guidelines in the face of a wide variety of CHIs. Therefore, in this study, we systematically evaluate and meta-analyze the overall efficacy and safety of CHIs in combination with PBC for advanced NSCLC, providing evidence and guidance for clinical use. A graphic summary of the meta-analysis is shown in **Figure 1**.

## 2 Methods

In this study, systematic review and meta-analysis were conducted in accordance with the Preferred Reporting Project for Systematic Evaluation and Meta-Analysis Protocols (PRISMA-P) (13). The protocol has been registered with the International Platform of Registered Systematic Review and Meta-analysis Protocols (INPLASY) under registration number: INPLASY202210104 (https://inplasy.com/inplasy-2022-1-0104/). Because all studies are published articles, ethical approval was not required.

### 2.1 Inclusion criteria

#### 2.1.1 Type of study

Only randomized controlled studies (RCTs) were included in this study to compare the efficacy and safety of CHI–first-line platinum-based chemotherapy combination (treatment group) and first-line platinum-based chemotherapy alone (control group) in the treatment of NSCLC.

#### 2.1.2 Types of participants

All patients were cytologically or pathologically confirmed cases of NSCLC and belonged to Stage III or IV according to American Joint Committee on Cancer Staging System (8th edition).

#### 2.1.3 Types of interventions

The control group was treated with PBC. The treatment group was treated with at most one CHI plus PBC regimen. The chemotherapy regimen was the same in the treatment and control groups, and no restrictions were placed on the type, dose, and duration of chemotherapy drugs or CHI. CHIs included Aidi injection (drug approval number: Z52020236); Compound Kushen injection (drug approval number: Z14021231); Huachansu injection (drug approval number: Z34020274); Javanica oil emulsion injection (drug approval number: Z44021325); Kanglaite injection (drug approval number: Z10970091); Kangai injection (drug approval number: Z20026868); Lanxiangxi injection (drug approval number: H20110114); Lentinan injection (drug approval number: H20030131); Shenfu injection (drug approval number: Z51020664); Shenmai injection (drug approval number: Z13020888); Shenqifuzheng injection (drug approval number: Z19990065); Xiaoaiping injection (drug approval number: Z20025868); Huangqi injection (drug approval number: Z23020782); and Chansu injection (drug approval number: Z34020604). These CHIs have been approved by China’s State Food and Drug Administration for cancer treatment (14). PBC included NP (vinorelbine plus cisplatin), PP (paclitaxel/albumin paclitaxel/paclitaxel liposome plus cisplatin), PC (paclitaxel/albumin paclitaxel liposome plus carboplatin), GP (gemcitabine plus cisplatin), GC (gemcitabine plus carboplatin), DP (docetaxel plus cisplatin), DC (docetaxel plus carboplatin), AP (pemetrexed plus cisplatin), and AC (pemetrexed plus carboplatin).

#### 2.1.4 Types of outcome measures

According to World Health Organization (WHO) (15) guidelines for solid tumor responses or Response Evaluation Criteria in Solid Tumors (RECIST) (16), tumors were evaluated as complete response (CR), partial response (PR), stable disease (SD), and progressive disease (PD). The primary outcomes were objective response rate (ORR) and disease control rate (DCR). ORR refers to the proportion of patients with CR or PR. DCR refers to the proportion of patients with CR, PR, or SD. Secondary outcomes were QoL and toxic side effects. QoL was assessed according to the Karnofsky performance scale (KPS) (17). An increase or decrease of 10 points was considered an improvement in QoL (18). The safety indexes were myelosuppression and digestive tract reaction. Standard Classification of WHO or National Cancer Institute Common Terminology Criteria for Adverse Events (NCI-CTCAE) was used to measure the incidence of leukopenia, decreased hemoglobin, thrombocytopenia, nausea and vomiting, diarrhea, and constipation. The involved studies included at least one primary outcome.

### 2.2 Exclusion criteria

The studies 1) which included animal experiments, literature reviews, case reports, and other unrelated studies; 2) in which patients received multiple CHIs or non-PBC regimens simultaneously; 3) in which ORR or DCR was not evaluated in the literature; 4) that included single-arm trials; 5) for which data were not available and those for which data were still not available after contacting the author by email; and 6) where no references to specific randomization methods or to “high risk” assessments of risk bias were made were excluded from this meta-analysis.

### 2.3 Information sources

PubMed, EMBASE, Cochrane, China National Knowledge Infrastructure (CNKI), Wanfang Data, VIP Database for Chinese Technical Periodicals (VIP), and SinoMed were searched systematically from their inception until July 1, 2023. The language of RCTs was limited to Chinese and English.

### 2.4 Search strategy

The literature search was conducted by combining subject words and free words. To ensure a comprehensive search, the search terms of CHIs and NSCLC were mainly formulated as follows (1) NSCLC, including “Carcinoma,” “non-small-cell Lung,” “Lung Carcinoma,” “Non-Small-Cell,” “Non-Small-Cell Lung Carcinomas,” “Non-small Cell Lung Carcinoma,” “Non-Small Cell Lung Cancer,” and “NSCLC.” (2) CHIs not only include the overall name, such as “Chinese herbal injection,” “Chinese medicine injection,” “injection of TCM,” but also contains the name of the specific injection, such as “Aidi,” “Chansu,” “Compound Kushen,” “Huachansu,” “Xiaoaiping,” “Kanglaite,” “Javanica oil emulsion,” “Shenqifuzheng,” “Kangai,” “Shenfu,” “Huangqi,” “Lentinan,” “Shenmai,” and “Lanxiangxi.” More detailed search terms and search policies are provided in **Supplementary Materials**.

### 2.5 Study selection

The retrieved literature were imported into Notexpress (version 3.0) for literature management. Two researchers (QC and WZ) independently screened the literature according to inclusion and exclusion criteria. First, literature management software deleted duplicate works of literature, and the literature that evidently did not meet the inclusion criteria were deleted by reading the title and abstract of the remaining literature. The literature obtained after the preliminary screening were downloaded and read. The final screening was conducted according to the type of research, type of patients, intervention measures, and outcome indicators. In case of any disagreement in the screening process, a consensus was reached through discussion with Hou Wei.

### 2.6 Data extraction

Two researchers (DW and CQ) used Microsoft Excel 2019 to independently extract data, including 1) publication year, first author, publishing country, and region; 2) the random model and implementation method and the blind method; 3) number of subjects, age, sex, and pathological type; and 4) specific drugs, dosage, course of treatment and duration of treatment.

### 2.7 Risk of bias and quality assessment

Two researchers (KC and SH) independently evaluated literature quality using the “Risk of Bias Assessment Tool” in the Cochrane Handbook for Randomized Controlled Trials (19). The evaluation mainly focused on seven aspects of randomization, assignment concealment, patient or investigator blindness, outcome evaluator blindness, outcome data integrity, selective outcome reporting, and other bias, and the evaluation results were categorized into “low risk,” “unclear,” or “high risk.” In case of any disagreement, the study team discussed it with HW to decide the result. The Grading of Recommendations Assessment, Development, and Evaluation (GRADE) was used to evaluate the quality of evidence, focusing on five aspects of downgrade factors (risk of bias, inconsistency, indirectness, imprecision, and publication bias) and three elements of upgrade factors (large effect, plausible confounding, and dose-response gradient). The evaluation results of downgrade factors were categorized into “not serious,” “serious,” and “very serious.” The evaluation results of upgrade factors were categorized into “no,” “large,” and “very large.” The two factors were combined to evaluate the quality of evidence as high, medium, low, or very low (20).

### 2.8 Statistical methods and analysis

In this study, Review Manager software (version 5.4) was used for meta-analysis. The dichotomous variables were expressed by risk ratio (RR) with 95% confidence intervals (CIs). A P-value < 0.05 was considered statistically significant.

Considering the potential heterogeneity between the trials, I^2^ and P-values were used to I^2^ was used to quantitate the heterogeneity; if studies or subgroups with P>0.1, and I^2^<50% were included, a meta-analysis using fixed effect models showed little to no heterogeneity between studies, and if studies or subgroups with P<0.1 and I^2^>50%, respectively, were included, it was indicative of a large heterogeneity among the included studies. By eliminating the studies one by one, we determined the literature with high heterogeneity and analyzed the causes of heterogeneity. In cases where heterogeneity of the literatures with high heterogeneity was significantly reduced after removal and the evaluation results were not affected, the literature was retained, and the random effects model were used for analysis (21). Descriptive analysis was performed if significant clinical heterogeneity was observed even after removal. Simultaneously, we conducted subgroup analysis according to the type of CHIs, and only descriptive analysis was performed when there were less than three included studies. In case of more than ten studies, a funnel plot was used to assess publication bias (22).

### 2.9 Sensitivity analysis

To ensure the stability of the meta-analysis results, we performed sensitivity analyses of the primary outcome measures by excluding articles by screening for sample size, duration of treatment for CHIs, and year of publication.

### 2.10 Trial sequential analysis

TSA software version 0.9 was used for sequential analysis of the primary outcome indicators, ORR and DCR, to reduce false positive results caused by random error, estimate the amount of information needed for meta-analysis, and further improve the credibility of this study.

## 3 Results

### 3.1 Study selection

**Figure 2:**
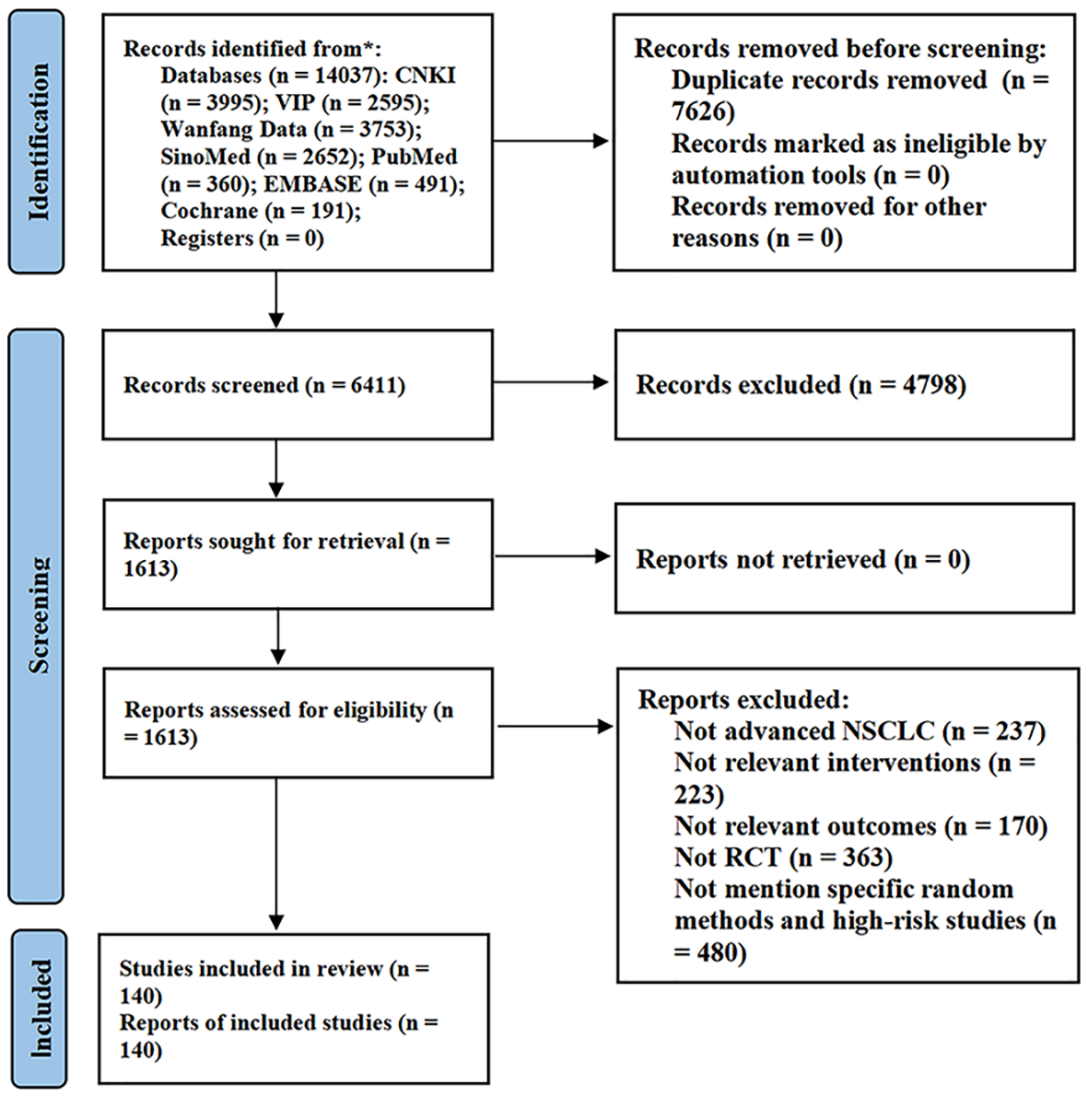
Flow diagram of study selection. CNKI, China National Knowledge Infrastructure; VIP, Chinese Scientific Journal Database.

A total of 14037 articles were retrieved from the database. First, 7626 duplicate articles were removed, and the titles and abstracts of the remaining 6411 articles were scanned. Of these, 4798 were removed, because they did not meet the inclusion and exclusion criteria. Further, the remaining 1613 articles were further screened and evaluated, and 993 articles were excluded. Finally, owing to a large number of clinical studies in this field, we excluded low-quality literature with large risk of bias in order to ensure the quality of evidence of meta-analysis. Through quality evaluation of the remaining 620 articles, 480 studies that did not mention specific randomized methods and literature quality evaluation as “high risk” were excluded. A total of 140 studies were included in the meta-analysis (23–162). The detailed literature screening process is shown in **Figure 2**.

### 3.2 Study characteristics

The baseline characteristics of the included studies are detailed in **Supplementary Table 1**. The 140 studies had a total of 12053 patients, including 6083 in the treatment group, 5970 in the control group, and 3912, 4976, and 837 in squamous cell carcinoma, adenocarcinoma, and other NSCLC pathological types, respectively; the number of subjects in each study ranged from 24 to 96, and the age range varied from 25 to 87.

All RCTs were conducted in China, and the specific numbers of the 12 CHIs included were as follows: Aidi injection was used in 31 trials (40, 43, 50, 53, 55, 58–60, 72, 76, 79, 85, 87–90, 93, 96, 101, 106, 108, 121, 125–127, 131, 141, 143, 151, 159, 161); Compound Kushen injection was used in 16 trials (44, 47, 54, 75, 97, 98, 113, 116, 130, 132, 142, 144, 148, 150, 152, 160); Huachansu injection was used in 5 trials (25, 27, 42, 65, 92); Javanica oil emulsion injection was used in 7 trials (29, 80, 95, 109, 112, 114, 137); Kanglaite injection was used in 21 trials (30, 33, 34, 36, 56, 63, 66, 67, 70, 94, 104, 105, 110, 118, 124, 133, 134, 136, 146, 147, 158); Kangai injection was used in 14 trials (31, 35, 48, 49, 51, 64, 71, 119, 120, 138, 140, 145, 149, 162); Lanxiangxi injection was used in 5 trials (39, 77, 103, 128, 157); Lentinan injection was used in 2 trials (86, 155); Shenfu injection was used in 2 trials (83, 117); Shenmai injection was used in 10 trials (26, 28, 32, 68, 74, 99, 102, 107, 129, 153); Shenqifuzheng injection was used in 18 trials (24, 38, 40, 45, 46, 57, 69, 81, 82, 84, 111, 115, 122, 123, 135, 139, 154, 156); and Xiaoaiping injection was used in 9 trials (23, 37, 52, 61, 62, 73, 78, 91, 100).

### 3.3 Quality evaluation

**Figure 3:**
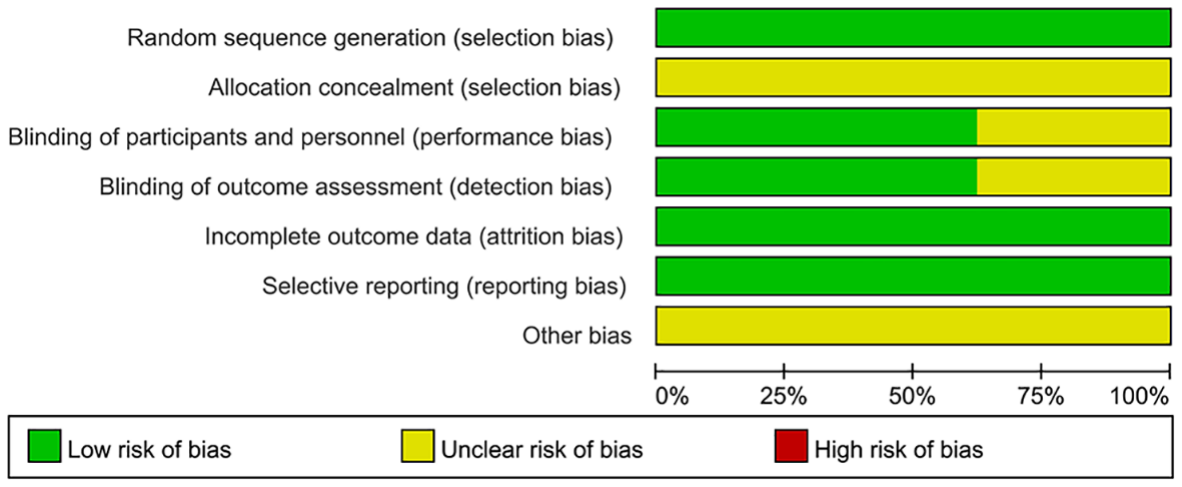
Risk of bias graph. Methodological quality evaluation of the included literature.

The methodological quality assessment of the included literature is detailed in **Figure 3** and **Supplementary Figure 1**. In terms of randomization methods, all 140 studies in the pooled analysis were rated as “low risk” owing to the exclusion of literature that did not mention specific randomization methods. All studies were classified as “unclear,” because they did not mention allocation concealment. All 53 studies that did not mention blinding and did not use a placebo were rated as “unclear,” because subjective outcome measures, including QoL, would be affected by blinding; however, 87 studies had objective outcome measures such as ORR and DCR and did not include subjective outcome measures. Clinical discrimination would not be affected by blinding and was rated as “low risk.” Concerning the completeness of outcome data, because the data for outcome measures were consistent with those assigned to randomization, the risk of incomplete data was low, and all studies were rated as “low risk”.

In the selective assessment, all studies were rated as “low risk,” because they reported outcomes as described in the methodology. Finally, other biases were not mentioned in all the studies and were evaluated as “unclear.” (163) The GRADE assessment results of this study and the reasons for the downgrade are listed in **Table 1**.

**Table 1:** The results of GRADE evaluation.

Quality assessment	Numbers of RCTs	Risk of bias	Inconsistency	Indirectness	Imprecision	Publication bias	Large effect	Plausible confounding	Dose response gradient	RR (95% CI)	Certainty of evidence
ORR	140	serious^a^	not serious (I^2 ^= 0%, P=1.00)	not serious	not serious	strongly suspected^d^	no	no	no	1.35 [1.30, 1.41]	LOW
DCR	138	serious^a^	not serious (I^2 ^= 12%, P=0.12)	not serious	not serious	strongly suspected^d^	no	no	no	1.15 [1.13, 1.18]	LOW
QoL	53	serious^a^	serious^b^ (I^2 ^= 35%, P=0.007)	not serious	not serious	strongly suspected^d^	no	no	no	1.29 [1.24, 1.33]	Very LOW
Leukopenia	77	serious^a^	serious^b^ (I^2 ^= 74%, P<0.00001)	not serious	not serious	strongly suspected^d^	no	no	no	0.69 [0.64, 0.75]	Very LOW
Anemia	42	serious^a^	serious^b^ (I^2 ^= 55%, P<0.0001)	not serious	not serious	strongly suspected^d^	no	no	no	0.68 [0.60, 0.77]	Very LOW
Thrombocytopenia	63	serious^a^	serious^b^ (I^2 ^= 48%, P<0.0001)	not serious	not serious	strongly suspected^d^	no	no	no	0.67 [0.61, 0.74]	Very LOW
Nausea and Vomiting	61	serious^a^	serious^b^ (I^2 ^= 72%, P<0.00001)	not serious	not serious	strongly suspected^d^	no	no	no	0.69 [0.63, 0.76]	Very LOW
Diarrhea	17	serious^a^	not serious (I^2 ^= 0%, P=0.85)	not serious	serious^c^	strongly suspected^d^	no	no	no	0.59 [0.48, 0.73]	Very LOW
Constipation	6	serious^a^	not serious (I^2 ^= 13%, P=0.33)	not serious	very serious^c^	strongly suspected^d^	no	no	no	0.68 [0.54, 0.86]	Very LOW

^a^Unclear description of the hidden methods of random allocation.

^b^Point estimates vary widely from study to study.

^c^The number of studies was too small and the confidence interval was too wide to be accurate.

^d^The funnel plots were asymmetrical, which indicated that publication bias might influence the results of the analysis.

ORR, objective response rate; DCR, disease control rate; QoL, quality of life; CI, confidence interval; RR, risk ratio.

### 3.4 Effectiveness and safety

The overall meta-analysis results are detailed in **Table 2**, and the subgroup analyses by type of CHIs are detailed in **Table 3**.

**Table 2:** Summary of the meta-analysis.

Outcomes	Studies	Participants	Statistical methods	Pooled RRs (95% CI)	P	Heterogeneity	
						I^2^	P_h_
ORR	140	12019	REM	1.35 [1.30, 1.41]	<0.00001	0%	1.00
DCR	138	11959	REM	1.15 [1.13, 1.18]	<0.00001	12%	0.12
QoL	53	4263	FEM	1.29 [1.24, 1.33]	<0.00001	35%	0.007
Leukopenia	77	6721	REM	0.69 [0.64, 0.75]	<0.00001	74%	<0.00001
Anemia	42	3341	REM	0.70 [0.62, 0.79]	<0.00001	58%	<0.00001
Thrombocytopenia	63	5056	REM	0.68 [0.62, 0.75]	<0.00001	51%	<0.00001
Nausea and Vomiting	61	5328	REM	0.69 [0.63, 0.76]	<0.00001	72%	<0.00001
Diarrhea	17	1674	FEM	0.59 [0.48, 0.73]	<0.00001	0%	0.85
Constipation	6	790	FEM	0.68 [0.54, 0.86]	0.001	13%	0.33

ORR, objective response rate; DCR, disease control rate; QoL, quality of life; FEM, fixed-effects model; REM, random-effects model; CI, confidence interval; RRs, risk ratios; P_h_, P-value for heterogeneity test.

**Table 3:** Subgroup analyses of all outcomes.

Subgroups	Number of studies	Pooled RRs (95% CI)	Z	P	Heterogeneity	
					I^2^	P_h_
ORR						
Aidi injection	31	1.40 [1.29, 1.52]	7.81	<0.00001	0%	1.00
Compound kushen injection	16	1.37 [1.23, 1.52]	5.69	<0.00001	0%	0.96
Huachansu injection	5	1.59 [1.21, 2.10]	3.28	0.001	0%	0.77
Javanica oil emulsion injection	7	1.20 [1.00, 1.44]	1.95	0.05	0%	0.83
Kanglaite injection	21	1.37 [1.22, 1.53]	5.42	<0.00001	0%	0.59
Kangai injection	14	1.38 [1.20, 1.59]	4.57	<0.00001	0%	0.98
Lanxiangxi injection	5	1.45 [1.08, 1.96]	2.44	0.01	57%	0.05
Shenmai injection	10	1.32 [1.14, 1.53]	3.73	0.0002	0%	0.88
Shenqifuzheng injection	18	1.28 [1.15, 1.43]	4.47	<0.00001	0%	0.53
Xiaoaiping injection	9	1.42 [1.20, 1.67]	4.20	<0.0001	0%	0.77
DCR						
Aidi injection	31	1.18 [1.14, 1.22]	8.89	<0.00001	0%	0.79
Compound kushen injection	16	1.18 [1.12, 1.24]	6.57	<0.00001	0%	0.92
Huachansu injection	5	1.30 [1.02, 1.65]	2.13	0.03	68%	0.01
Javanica oil emulsion injection	7	1.10 [1.00, 1.22]	1.90	0.06	45%	0.09
Kanglaite injection	20	1.13 [1.06, 1.21]	3.60	0.0003	40%	0.03
Kangai injection	14	1.18 [1.11, 1.26]	4.95	<0.00001	0%	0.53
Lanxiangxi injection	5	1.20 [1.02, 1.41]	2.23	0.03	65%	0.02
Shenmai injection	10	1.17 [1.09, 1.25]	4.48	<0.00001	0%	0.53
Shenqifuzheng injection	17	1.12 [1.06, 1.18]	4.09	<0.0001	22%	0.20
Xiaoaiping injection	9	1.12 [1.03, 1.20]	2.77	0.006	0%	0.94
QoL						
Aidi injection	9	1.26 [1.17, 1.37]	5.88	<0.00001	0%	0.67
Compound kushen injection	5	1.18 [1.08, 1.28]	3.69	0.0002	5%	0.38
Huachansu injection	4	1.14 [1.04, 1.25]	2.75	0.006	0%	0.63
Javanica oil emulsion injection	4	1.50 [1.27, 1.78]	4.71	<0.00001	0%	0.91
Kanglaite injection	4	1.17 [1.06, 1.29]	3.12	0.002	49%	0.12
Kangai injection	4	1.22 [1.09, 1.37]	3.40	0.0007	0%	0.89
Shenmai injection	4	1.41 [1.18, 1.69]	3.69	0.002	0%	0.41
Shenqifuzheng injection	10	1.56 [1.41, 1.73]	8.63	<0.00001	43%	0.07
Xiaoaiping injection	6	1.32 [1.17, 1.48]	4.56	<0.00001	0%	0.89
Leukopenia						
Aidi injection	18	0.77 [0.67, 0.88]	3.88	0.0001	67%	<0.0001
Compound kushen injection	8	0.57 [0.46, 0.71]	5.04	<0.00001	37%	0.13
Huachansu injection	4	0.78 [0.53, 1.13]	1.32	0.19	79%	0.003
Javanica oil emulsion injection	3	0.87 [0.74, 1.02]	1.75	0.08	0%	0.77
Kanglaite injection	7	0.80 [0.60, 1.08]	1.43	0.15	75%	0.0006
Kangai injection	6	0.58 [0.45, 0.76]	4.05	<0.0001	31%	0.20
Lanxiangxi injection	3	0.90 [0.71, 1.15]	0.84	0.40	80%	0.006
Shenmai injection	6	0.62 [0.52, 0.74]	5.33	<0.00001	0%	0.90
Shenqifuzheng injection	12	0.63 [0.55, 0.71]	7.38	<0.00001	0%	0.56
Xiaoaiping injection	7	0.73 [0.64, 0.84]	4.39	<0.0001	6%	0.38
Anemia						
Aidi injection	7	0.78 [0.66, 0.93]	2.73	0.006	0%	0.69
Compound kushen injection	3	0.50 [0.33, 0.77]	3.19	0.001	0%	0.40
Kanglaite injection	5	0.80 [0.57, 1.13]	1.26	0.21	69%	0.01
Kangai injection	3	0.62 [0.32, 1.20]	1.43	0.15	0%	0.59
Lanxiangxi injection	3	0.89 [0.74, 1.07]	1.27	0.20	22%	0.28
Shenmai injection	4	0.32 [0.18, 0.58]	3.81	0.0001	0%	0.94
Shenqifuzheng injection	8	0.55 [0.44, 0.68]	5.40	<0.00001	0%	0.78
Xiaoaiping injection	6	0.76 [0.65, 0.89]	3.49	0.0005	4%	0.39
Thrombocytopenia						
Aidi injection	16	0.71 [0.61, 0.81]	4.94	<0.00001	0%	0.93
Compound kushen injection	6	0.52 [0.38, 0.71]	4.11	<0.0001	17%	0.31
Huachansu injection	2	1.03 [0.57, 1.87]	0.09	0.92	61%	0.11
Kanglaite injection	6	0.78 [0.59, 1.05]	1.65	0.10	57%	0.04
Kangai injection	6	0.44 [0.29, 0.68]	3.72	0.0002	33%	0.19
Lanxiangxi injection	3	0.86 [0.58, 1.25]	0.80	0.42	65%	0.06
Shenmai injection	5	0.56 [0.33, 0.94]	2.19	0.03	58%	0.05
Shenqifuzheng injection	10	0.78 [0.66, 0.93]	2.78	0.005	0%	0.49
Xiaoaiping injection	6	0.65 [0.53, 0.79]	4.42	<0.00001	0%	0.43
Nausea and Vomiting						
Aidi injection	12	0.82 [0.70, 0.98]	2.21	0.03	73%	<0.0001
Compound kushen injection	5	0.73 [0.61, 0.86]	3.61	0.0003	22%	0.28
Kanglaite injection	9	0.62 [0.42, 0.90]	2.48	0.01	83%	<0.00001
Kangai injection	8	0.57 [0.45, 0.72]	4.69	<0.00001	0%	0.84
Lanxiangxi injection	3	0.96 [0.74, 1.23]	0.35	0.73	68%	0.04
Shenmai injection	7	0.62 [0.53, 0.73]	5.88	<0.00001	0%	0.57
Shenqifuzheng injection	6	0.51 [0.36, 0.73]	3.66	0.0003	70%	0.005
Xiaoaiping injection	5	0.75 [0.60, 0.92]	2.69	0.007	13%	0.33
Diarrhea						
Compound kushen injection	5	0.56 [0.40, 0.77]	3.50	0.0005	0%	0.84
Constipation						
Compound kushen injection	3	0.66 [0.49, 0.89]	2.76	0.006	49%	0.14

ORR, objective response rate; DCR, disease control rate; QoL, quality of life; CI, confidence interval; RRs, risk ratios; P_h_, P-value for heterogeneity test.

#### 3.4.1 Objective response rate

**Figure 4:**
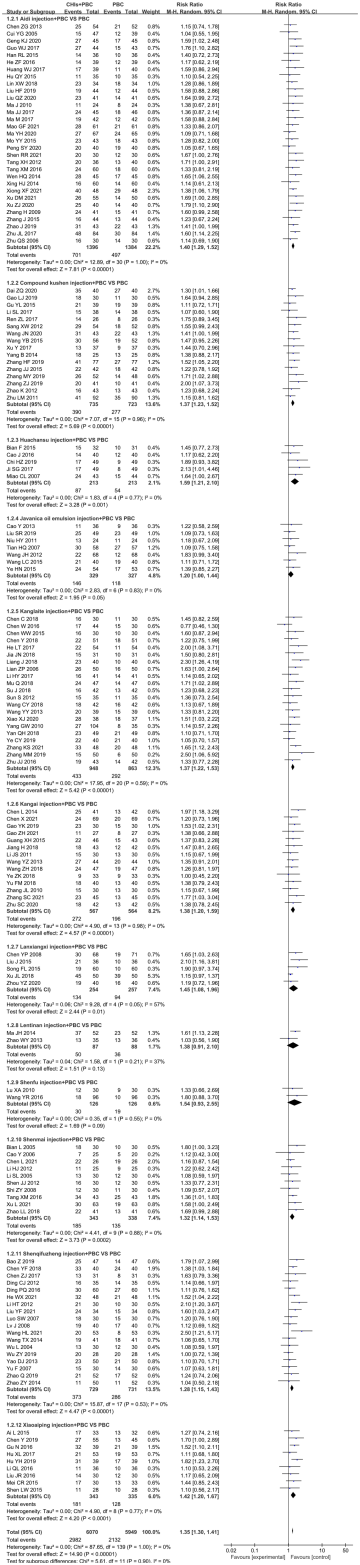
Forest plot of ORR in PBC versus PBC plus CHIs. The meta-analysis results, stratified by the type of CHIs, showed differences in ORR between PBC and PBC plus CHIs. Objective response rate ORR=(CR+PR)/total cases×100%; CHIs, Chinese herbal injections; PBC, platinum-based chemotherapy.

A total of 140 RCTs including 12 CHIs reported ORR outcomes. No statistical heterogeneity was found after pooling (I^2^=0%, P=1.00); however, the heterogeneity of Lanxiangxi injection subgroup was high (I^2^=57%, P=0.05). Therefore, the random effect model was used for meta-analysis. The results showed that the ORR of the treatment group was significantly higher than that of the control group (RR=1.35, 95% CI: 1.30–1.41, P<0.001; **Figure 4**). According to the results of subgroup analysis of CHIs type, Aidi injection subgroup (RR=1.40, 95% CI: 1.29–1.52, P<0.001; I^2^=0%), Compound Kushen injection subgroup (RR=1.37, 95% CI: 1.23–1.52, P<0.001; I^2^=0%), Huachansu injection subgroup (RR=1.59, 95% CI: 1.21–2.10, P=0.001; I^2^=0%, Kanglaite injection subgroup (RR=1.37, 95% CI: 1.22–1.53, P<0.001; I^2^=0%), Kangai injection subgroup (RR=1.38, 95% CI: 1.20–1.59, P<0.001; I^2^=0%), Lanxiangxi injection subgroup (RR=1.45, 95% CI: 1.08–1.96, P=0.01; I^2^=57%), Shenmai injection subgroup (RR=1.32, 95% CI: 1.14–1.53, P<0.001; I^2^=0%), Shenqifuzheng injection subgroup (RR=1.28, 95% CI: 1.15–1.43, P<0.001; I^2^=0%), Xiaoaiping injection subgroup (RR=1.42, 95% CI: 1.20–1.67, P<0.001; I^2^=0%) showed that the ORR of the treatment group was significantly higher than that of the control group. However, Javanica oil emulsion injection subgroup (RR=1.20, 95% CI: 1.00–1.44, P=0.05; I^2^=0%) exhibited no significant difference compared to the control group in terms of ORR. After individually selecting studies, it was found that the heterogeneity of Lanxiangxi injection subgroup mainly arose from one trial (128), which could be attributed to the low dose of Lanxiangxi injection in this study (400 mg/d, 7 days/course, 4 courses). After excluding this trial, there was no significant difference between the merged statistical values and the original results (RR=1.61, 95% CI: 1.23–2.11, P=0.001; I^2^=0%). Less than two articles were retrieved for the Lentinan and Shenfu injection subgroups, so only descriptive analysis was performed without merging. Overall, Lentinan and Shenfu injection subgroups did not exhibit any significant advantage in terms of ORR.

#### 3.4.2 Disease control rate

**Figure 5:**
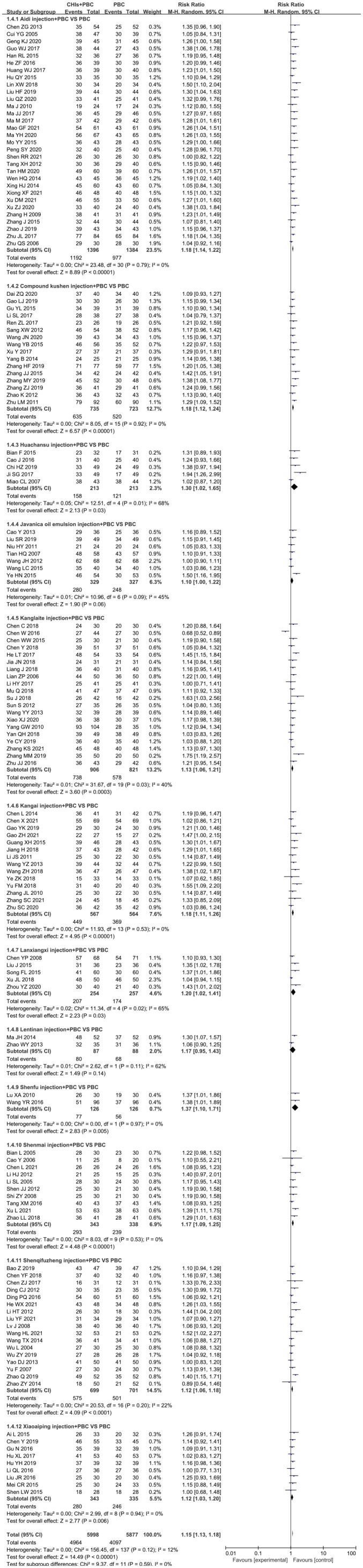
Forest plot of DCR in PBC versus PBC plus CHIs. The meta-analysis results, stratified by the type of CHIs, showed differences in DCR between PBC and PBC plus CHIs. Disease control rate DCR=(CR+PR+SD)/total cases×100%; CHIs, Chinese herbal injections; PBC, platinum-based chemotherapy.

A total of 138 RCTs covering 12 CHIs reported the outcomes of DCR. No significant heterogeneity was found in the combined analysis (I^2^=12%, P=0.12). However, the heterogeneity of the Huachansu injection subgroup (I^2^=68%, P=0.01) and Lanxiangxi injection subgroup (I^2^=65%, P=0.02) was relatively high. Therefore, the random effect model was used for meta-analysis. The results showed that the DCR of the treatment group was significantly higher than that of the control group (RR=1.15, 95% CI: 1.13–1.18, P<0.001; **Figure 5**). Subgroup analysis according to the type of CHIs showed that the DCR value of Aidi injection subgroup (RR=1.18, 95% CI: 1.14–1.22, P<0.001; I^2^=0%), Compound Kushen injection subgroup (RR=1.18, 95% CI: 1.12–1.24, P<0.001; I^2^=0%), Huachansu injection subgroup (RR=1.30, 95% CI: 1.02–1.65, P=0.03; I^2^=68%), Kanglaite injection subgroup (RR=1.13, 95% CI: 1.06–1.21, P<0.001; I^2^=40%), Kangai injection subgroup (RR=1.18, 95% CI: 1.11–1.26, P<0.001; I^2^=0%), Lanxiangxi injection subgroup (RR=1.20, 95% CI: 1.02–1.41, P=0.01; I^2^=65%), Shenmai injection subgroup (RR=1.17, 95% CI: 1.09–1.25, P<0.001; I^2^=0%), Shenqifuzheng injection subgroup (RR=1.12, 95% CI: 1.06–1.25, P<0.001; I^2^=22%), and Xiaoaiping injection subgroup (RR=1.12, 95% CI: 1.03–1.20, P=0.01; I^2^=0%) showed that the DCR of the treatment group was significantly higher than that of the control group. However, Javanica oil emulsion injection subgroup (RR=1.10, 95% CI: 1.00–1.22, P=0.06; I^2^=45%) exhibited no significant difference compared to the control group in terms of DCR. The heterogeneity of the Huachansu and Lanxiangxi injection subgroups arose from two trials (Miao CL, 2017 (128) respectively). The heterogeneity may be due to the lower dosage of CHIs compared with that in other studies (Huachansu injection: 20 mL/d, 5 days/course, 3–6 courses; Lanxiangxi injection: 400 mg/d, 7 days/course, 4 courses). After excluding the literature of Miao CL (2017) and (128), the statistical values were as follows: Huachansu injection subgroup, RR=1.39, 95% CI: 1.16–1.67, P<0.001; I^2^=4%; Lanxiangxi injection subgroup, RR=1.24, 95% CI: 1.08–1.42, P=0.002; I^2^=15%. No significant differences were observed with respect to the original results. Less than two articles were retrieved on the Lentinan and Shenfu injection subgroups; hence, only descriptive analysis was performed without merging. Altogether, Shenfu injection subgroup had a positive effect on DCR. However, the Lentinan injection subgroup exhibited no obvious advantage in terms of DCR.

#### 3.4.3 Quality of life

**Figure 6:**
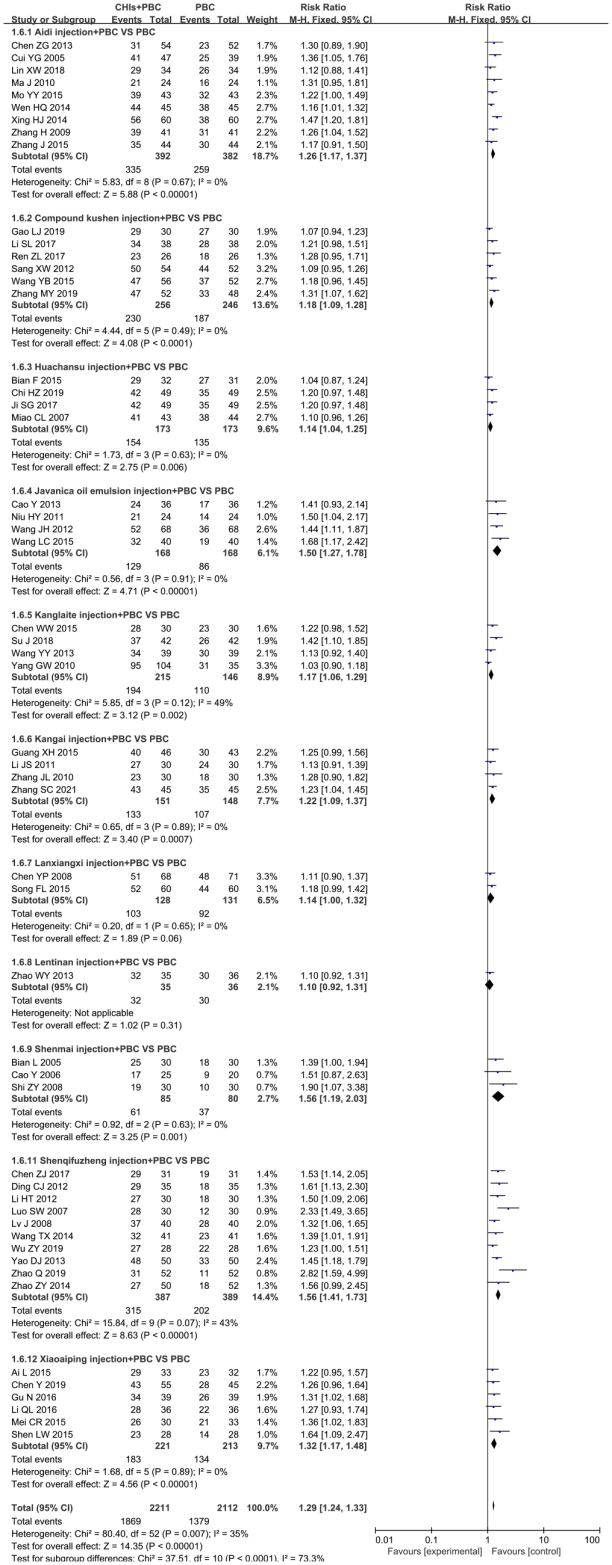
Forest plot of QoL in PBC versus PBC plus CHIs. The meta-analysis results, stratified by the type of CHIs, showed differences in QoL between PBC and PBC plus CHIs. QoL, quality of life; CHIs, Chinese herbal injections; PBC, platinum-based chemotherapy.

A total of 52 studies determined QoL and included 11 CHIs, with low heterogeneity after combined analysis (I^2^=35%, P=0.01). Therefore, the fixed effects model was used for meta-analysis. The results showed that the QoL of the treatment group was significantly higher than that of the control group (RR=1.29, 95% CI: 1.24–1.33, P<0.001; **Figure 6**). Additionally, the subgroup analysis showed that QoL of Aidi injection subgroup (RR=1.26, 95% CI: 1.17–1.37, P<0.001; I^2^=0%), Compound Kushen injection subgroup (RR=1.18, 95% CI: 1.09–1.28, P<0.001; I^2^=0%), Huachansu injection subgroup (RR=1.14, 95% CI: 1.04–1.25, P=0.01; I^2^=0%), Javanica oil emulsion injection subgroup (RR=1.50, 95% CI: 1.27–1.78, P<0.001; I^2^=0%), Kanglaite injection subgroup (RR=1.42, 95% CI: 1.27–1.59, P=0.002; I^2^=49%), Kangai injection subgroup (RR=1.22, 95% CI: 1.09–1.37, P=0.001; I^2^=0%), Shenmai injection subgroup (RR=1.56, 95% CI: 1.19– 2.03, P=0.001; I^2^=0%), Shenqifuzheng injection subgroup (RR=1.56, 95% CI: 1.41–1.73, P<0.001; I^2^=43%), and Xiaoaiping injection subgroup (RR=1.32, 95% CI: 1.17–1.48, P<0.001; I^2^=0%) were increased significantly. The literature search of the Lanxiangxi injection subgroup yielded less than two articles; hence, only descriptive analysis was carried out without merging. Overall, Lanxiangxi injection subgroup exhibited no obvious advantage in terms of QoL.

#### 3.4.4 Leukopenia

A total of 77 studies including 12 CHIs reported the occurrence of leukopenia. The heterogeneity of the combined analysis was high (I^2^=75%, P<0.001). Therefore, a random-effects model was used for meta-analysis. The results showed that the incidence of leukopenia in the treatment group was significantly lower than that in the control group (RR=0.69, 95% CI: 0.64–0.75, P<0.001). By individually eliminating the literature, it was found that the heterogeneity mainly arose from (39, 43, 65, 103) Tan XM (2020) (125, 158, 160, 161) and was significantly reduced after exclusion (I^2 ^= 13%, P=0.19). The heterogeneity may be related to the drug toxicity, intervention dose, course of treatment, and chemotherapy regimen of different CHIs. After excluding the literature with large heterogeneity, the pooled analysis was performed, and the statistical values were not significantly different from the original results (RR=0.68, 95% CI: 0.64–0.72, P<0.001). Further subgroup analysis showed that the incidence of leukopenia of Aidi injection subgroup (RR=0.77, 95% CI: 0.67–0.88, P<0.001; I^2^=67%), Compound Kushen injection subgroup (RR=0.57, 95% CI: 0.46–0.71, P<0.001; I^2^=37%), Kangai injection subgroup (RR=0.58, 95% CI: 0.45–0.76, P<0.001; I^2^=31%), Shenmai injection subgroup (RR=0.62, 95% CI: 0.52–0.74, P<0.001; I^2^=0%), Shenqifuzheng injection subgroup (RR=0.63, 95% CI: 0.55–0.71, P<0.001; I^2^=0%), and Xiaoaiping injection subgroup (RR=0.73, 95% CI: 0.64–0.84, P<0.001; I^2^=6%) was significantly decreased. However, Huachansu injection subgroup (RR=0.78, 95% CI: 0.53–1.13, P=0.19; I^2^=79%), Javanica oil emulsion injection subgroup (RR=0.87, 95% CI: 0.74–1.02, P=0.08; I^2^=0%), Kanglaite injection subgroup (RR=0.80, 95% CI: 0.60–1.08, P=0.15; I^2^=75%), and Lanxiangxi injection subgroup (RR=0.90, 95% CI: 0.71–1.15, P=0.40; I^2^=80%) exhibited no obvious advantage in terms of the incidence of leukopenia. The subgroup analysis showed that the heterogeneity of the Aidi injection subgroup was derived from (43), Tan XM (2020) (125), and (161) and that of the Huachansu injection subgroup was derived from (65). The heterogeneity of the Kanglaite injection subgroup was derived from (158) and that of the Lanxiangxi injection subgroup was derived from (157). The analysis found that the heterogeneity of the Aidi injection subgroup was derived from (43, 125), used NP chemotherapy regimen and did not specify the pathological type of the enrolled population (161), used a DP chemotherapy regimen, and Tan XM (2020) used a shorter intervention time for Aidi injection, with only 10 days for each course of treatment. The heterogeneity of the Huachansu injection subgroup was attributed to the small proportion of squamous cell carcinoma and adenocarcinoma in (65). The heterogeneity of the Kanglaite injection subgroup was derived from (158), in which the Kanglaite injection intervention duration was 10 days, which was relatively short. The heterogeneity of the Lanxiangxi injection subgroup was attributed to the pathological type of the included population in (157), which included only adenocarcinoma. After excluding the literature with large heterogeneity, the combined statistical values were as follows: Aidi injection subgroup, RR=0.68, 95% CI: 0.59^–^0.78, P<0.001; I^2^=12; Lanxiangxi injection subgroup, RR=0.99, 95% CI: 0.92^–^1.06, P=0.70; I^2^=0%. No significant differences were observed with respect to the original results. However, the Huachansu injection subgroup (RR=0.66, 95% CI: 0.54–0.80, P<0.001; I^2^=0%) excluded (65), while the Kanglaite injection subgroup (RR=0.74, 95% CI: 0.58–0.94, P=0.01; I^2^=36%) excluded (158). After excluding the above studies, the combined analysis showed that the value differed from the original result, and the literature heterogeneity had a certain effect on the meta-analysis results. After excluding heterogeneity, it was confirmed that the Huachansu and Kanglaite injections when combined with PBC were effective than the PBC alone in the alleviation of the incidence of leukopenia. The number of articles retrieved for the Shenfu injection subgroup was less than two; hence, only descriptive analysis was performed without merging. Overall, the Shenfu injection could play a positive role in the alleviation of leukopenia. Forest plot of leukopenia is provided in the **Supplementary Figure 2**.

#### 3.4.5 Anemia

A total of 42 studies including 8 CHIs reported the occurrence of anemia. The heterogeneity of the combined analysis was high (I^2^=58%, P<0.001); hence, the random-effects model was used for the meta-analysis. The results showed that the incidence of anemia in the treatment group was significantly lower than that in the control group (RR=0.70, 95% CI: 0.62–0.79, P<0.00001). By paying attention to the exclusion literature, it was found that the heterogeneity mainly arose from the literature of (39, 65, 73, 103, 118, 158) and was significantly reduced after exclusion (I^2^=0%, P=0.58). The intervention measures of these studies were found to be different, which may be related to the drug toxicity, intervention dose, course of treatment, and chemotherapy regimen of different CHIs. After excluding studies with large heterogeneity, the statistical values were not significantly different from the original results (RR=0.65, 95% CI: 0.59–0.71, P<0.001; I^2^=0%). Further subgroup analysis showed that The incidence of anemia of the Aidi injection subgroup (RR=0.78, 95% CI: 0.66–0.93, P=0.01; I^2^=0%), Compound Kushen injection subgroup (RR=0.50, 95% CI: 0.33–0.77, P<0.001; I^2^=0%), Shenmai injection subgroup (RR=0.32, 95% CI: 0.18–0.58, P<0.001; I^2^=0%), Shenqifuzheng injection subgroup (RR=0.55, 95% CI: 0.44–0.68, P<0.001; I^2^=0%), and Xiaoaiping injection subgroup (RR=0.76, 95% CI: 0.65–0.89, P=0.001; I^2^=4%) was significantly reduced. However, Kanglaite injection subgroup (RR=0.80, 95% CI: 0.57–1.13, P=0.21; I^2^=69%), Kangai injection subgroup (RR=0.62, 95% CI: 0.32–1.20, P=0.15; I^2^=0%), and Lanxiangxi injection subgroup (RR=0.89, 95% CI: 0.74–1.07, P=0.20; I^2^=22%) exhibited no evident advantage in terms of the incidence of anemia. The heterogeneity of the Kanglaite injection subgroup was derived from (146), a study which did not specifically describe CHI and the cycle of chemotherapy and was considered as the main source of heterogeneity. The pooled statistics by excluding this literature showed no significant difference from the original results (RR=0.92, 95% CI: 0.72–1.16, P=0.46; I^2^=44%). Forest plot of anemia is provided in the **Supplementary Figure 3**.

#### 3.4.6 Thrombocytopenia

The occurrence of thrombocytopenia was reported in 63 studies including 8 CHIs. The heterogeneity was high in the pooled analysis (I^2^=51%, P<0.001); hence, the random-effects model was used for the meta-analysis. The results showed that the incidence of anemia in the treatment group was significantly lower than that in the control group (RR=0.68, 95% CI: 0.62–0.75, P<0.001). By individually excluding studies, we found that the heterogeneity mainly arose from (103, 117, 158, 161). After excluding these studies, the heterogeneity was significantly reduced (I^2^=19%, P=0.11). This may be related to the different toxicity, dose, intervention course, and chemotherapy regimens of Lanxiangxi, Shenfu, Kanglaite, and Aidi injections. After excluding studies with large heterogeneity, the pooled statistical values were not significantly different from the original results (RR=0.70, 95% CI: 0.64–0.76, P<0.001; I^2^=19%). Subgroup analysis showed that the incidence of thrombocytopenia of the Aidi injection subgroup (RR=0.71, 95% CI: 0.61–0.81, P<0.00001; I^2^=0%), Compound Kushen injection subgroup (RR=0.52, 95% CI: 0.38–0.71, P<0.0001; I^2^=17%), Kangai injection subgroup (RR=0.44, 95% CI: 0.29–0.68, P=0.0002; I^2^=33%), Shenmai injection subgroup (RR=0.56, 95% CI: 0.33–0.94, P=0.03; I^2^=58%), Shenqifuzheng injection subgroup (RR=0.78, 95% CI: 0.66–0.93, P=0.005; I^2^=0%), and Xiaoaiping injection subgroup (RR=0.65, 95% CI: 0.53–0.79, P<0.00001; I^2^=0%) was significantly reduced. However, Kanglaite injection subgroup (RR=0.78, 95% CI: 0.59–1.05, P=0.10; I^2^=57%) and Lanxiangxi injection subgroup (RR=0.86, 95% CI: 0.58–1.25, P=0.42; I^2^=65%) exhibited no obvious advantage in terms of the incidence of thrombocytopenia. In the subgroup analysis, the heterogeneity of the Shenmai injection subgroup was derived from (107) and that of the Kanglaite injection subgroup was derived from (146). The heterogeneity of the Lanxiangxi injection subgroup arose from (157). Through analysis, it was found that the heterogeneity of the Shenmai injection subgroup was due to the only pathological type of adenocarcinoma in the population included in (107). The heterogeneity of the Kanglaite injection subgroup was because (146) did not specifically describe CHI and the cycle of chemotherapy. The heterogeneity of the Lanxiangxi injection subgroup arose from the fact that the pathological type of the included population in (157) was only adenocarcinoma. After excluding the literature with large heterogeneity, the combined statistical values were as follows: Shenmai injection subgroup, RR=0.47, 95% CI: 0.27–0.84, P=0.05; I^2^=28%; Kanglaite injection subgroup, RR=0.86, 95% CI: 0.72–1.04, P=0.12; I^2^=24%; and Lanxiangxi injection subgroup, RR=0.93, 95% CI: 0.82–1.06, P=0.29; I^2^=0%. Literature heterogeneity within subgroups had no significant effect on the study results. Less than two articles were retrieved for the Shenfu injection subgroup; hence, only descriptive analysis was performed without pooling. Overall, the Shenfu injection subgroup could play a positive role in thrombocytopenia. Forest plot of thrombocytopenia is provided in the **Supplementary Figure 4**.

#### 3.4.7 Nausea and vomiting

A total of 61 RCTs including 12 CHIs reported on nausea and vomiting. The pooled heterogeneity was high (I^2^=72%, P<0.001); hence, the random-effects model was used for the meta-analysis. The results showed that the incidence of nausea and vomiting in the treatment group was significantly lower than that in the control group (RR=0.69, 95% CI: 0.63–0.76, P<0.001). The heterogeneity of the literature may be due to the different side effects of nausea and vomiting caused by different CHIs and chemotherapy regimens. By excluding (39, 43, 82, 103) Tan XM (2020), and (125), the heterogeneity of (158) and (161) was significantly reduced (I^2^=20%, P=0.11; RR=0.67, 95% CI: 0.62–0.72, P<0.001). The heterogeneity of the literature had little effect on the meta-analysis, and no significant difference was observed with respect to the original results. Subgroup analysis by CHIs type showed that the incidence of nausea and vomiting of the Aidi injection subgroup (RR=0.82, 95% CI: 0.70–0.98, P=0.03; I^2^=73%), Compound Kushen injection subgroup (RR=0.73, 95% CI: 0.61–0.86, P=0.003; I^2^=22%), Kanglaite injection subgroup (RR=0.62, 95% CI: 0.42–0.90, P=0.01; I^2^=83%), Kangai injection subgroup (RR=0.57, 95% CI: 0.45–0.72, P<0.001; I^2^=0%), Shenmai injection subgroup (RR=0.62, 95% CI: 0.53–0.73, P<0.001; I^2^=0%), Shenqifuzheng injection subgroup (RR=0.51, 95% CI: 0.36–0.73, P<0.001; I^2^=70%), and Xiaoaiping injection subgroup (RR=0.75, 95% CI: 0.06–0.92, P=0.01; I^2^=13%) were significantly reduced. However, Lanxiangxi injection subgroup (RR=0.96, 95% CI: 0.74–1.23, P=0.73; I^2^=68%) exhibited no obvious advantage in terms of the incidence of nausea and vomiting. Subgroup analysis showed that the heterogeneity of Aidi injection subgroup was derived from Ma JJ (2017) (89) and (121), that of the heterogeneity of Kanglaite injection subgroup was derived from (118, 158) the Shenqifuzheng injection subgroup was derived from (82) and (111), and that of the Lanxiangxi injection subgroup was derived from (157). According to the analysis, the heterogeneity of the Aidi injection subgroup was due to the relatively high proportion of males in the population included in Ma JJ (2017) (89, 121). Additionally, Ma JJ (2017) and (89) did not mention the pathological type of the included population. The heterogeneity of the Kanglaite injection subgroup was attributed to the fact that (118) only included patients in stage IIIb-IV and that (158) used antiemetic treatment before chemotherapy. The heterogeneity of the Shenqifuzheng injection subgroup arose from (82), in which all patients received antiemetics before chemotherapy. Moreover (111), did not mention the pathological type of the included population. The heterogeneity of the Lanxiangxi injection subgroup arose from the pathological type of the included population in (157), which was only adenocarcinoma. After excluding the literature with large heterogeneity, the combined statistical values were as follows: Kanglaite injection subgroup, RR=0.50, 95% CI: 0.39–0.65, P<0.001; I^2^=0%); Shenqifuzheng injection subgroup, RR=0.67, 95% CI: 0.56^–^0.81, P<0.001; I^2^=0%; and Lanxiangxi injection subgroup, RR=1.04, 95% CI: 0.94–1.15, P=0.50; I^2^=0%. In this study, the conclusions were not significantly different from the original results, and the literature heterogeneity within the subgroups had no significant effect on the study results. For the Aidi injection subgroup, the literature with large heterogeneity was excluded, and the random effects model was used to combine the results. The statistical value differed from the original result (RR=0.95, 95% CI: 0.88–1.03, P=0.23; I^2^=12%), and the literature heterogeneity had a certain impact on the meta-analysis. After excluding the heterogeneity, it was found that the Aidi injection exhibited no obvious advantage in terms of the incidence of nausea and vomiting between the treatment group and the control group. Because less than two articles were retrieved for the Javanica oil emulsion and Shenfu injection subgroups, only descriptive analysis was carried out without merging. Overall, the Shenfu injection subgroup positively affected the incidence of nausea and vomiting; however, the Javanica oil emulsion injection subgroup had no obvious advantage. Forest plot of nausea and vomiting is provided in the **Supplementary Figure 5**.

#### 3.4.8 Diarrhea

A total of 17 RCTs involving 10 CHIs reported outcomes for diarrhea. No statistical heterogeneity was found in the combined analysis (I^2^=0%, P=0.85); hence, the fixed effect model was used. The results showed that the incidence of diarrhea in the treatment group was significantly lower than that in the control group (RR=0.59, 95% CI: 0.48–0.73, P<0.001). Subgroup analysis according to the type of CHIs showed that the incidence of diarrhea was significantly lower in the Compound Kushen injection subgroup (RR=0.56, 95% CI: 0.40^–^0.77, P=0.001; I^2^=0%). Because less than two articles were retrieved for the Javanica oil emulsion and Xiaoaiping injection subgroups, only descriptive analysis was carried out without merging. Overall, the two had no apparent advantage in the incidence of diarrhea. Forest plot of diarrhea is provided in the **Supplementary Figure 6**.

#### 3.4.9 Constipation

A total of 6 RCTs covering 4 CHIs reported constipation outcomes. Little heterogeneity was observed in the combined analysis (I^2^=13%, P=0.33). The results showed that the incidence of constipation in the treatment group was significantly lower than that in the control group (RR=0.68, 95% CI: 0.54–0.86, P=0.001). The results of subgroup analysis according to CHIs type showed that the incidence of constipation was significantly reduced in the Compound Kushen injection subgroup (RR=0.66, 95% CI: 0.49–0.89, P=0.01; I^2^=49%). Forest plot of constipation is provided in the **Supplementary Figure 7**.

#### 3.4.10 Publication bias

**Figure 7:**
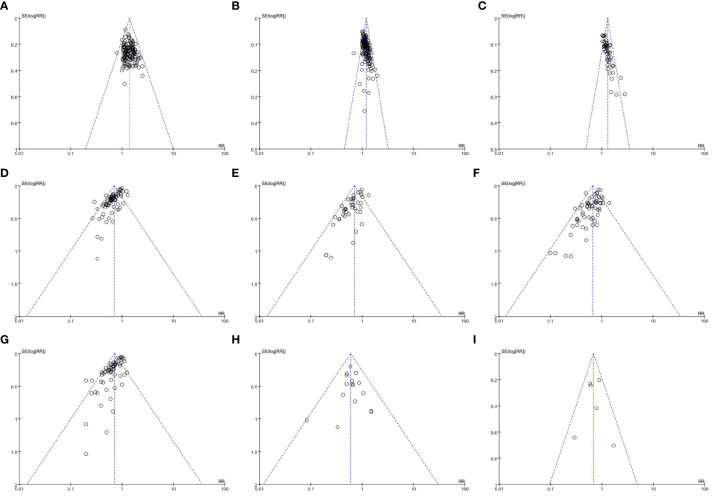
Funnel plots of outcomes. **(A)** objective response rate; **(B)** disease control rate; **(C)** quality of life; **(D)** leukopenia; **(E)** anemia; **(F)** thrombocytopenia; **(G)** nausea and vomiting; **(H)** diarrhea; **(I)** constipation.

**Figure 7** shows that the funnel plot based on ORR, DCR, QoL, leukopenia, anemia, thrombocytopenia, nausea and vomiting, diarrhea, and constipation is asymmetric, indicating a certain publication bias.

#### 3.4.11 Additional analyses

In terms of ORR, the pooled results of RCTs showed that the ORR of the treatment group was significantly higher than that of the control group (RR=1.35, 95% CI: 1.30–1.41, P<0.001). Sensitivity analysis showed that when only 32 RCTs with ≥50 participants (RR=1.34, 95% CI: 1.24–1.44, P<0.001), 31 RCTs with treatment duration ≥4 cycles (RR=1.35, 95% CI: 1.24–1.47, P<0.001), or 69 RCTs published within the last 5 years (RR=1.39, 95% CI: 1.32–1.46, P<0.001) were included, ORR only changed slightly from the previous results.

The pooled results of RCTs showed that the DCR of the treatment group was significantly higher than that of the control group (RR=1.15, 95% CI: 1.13–1.18, P<0.001). Sensitivity analysis showed that when only 32 RCTs with ≥50 participants (RR=1.18, 95% CI: 1.13–1.24, P<0.00001), 31 RCTs with treatment duration ≥4 cycles (RR=1.18, 95% CI: 1.11–1.25, P<0.001), or 68 RCTs published in the last 5 years (RR=1.18, 95% CI: 1.15–1.21, P<0.001) were included, DCR only changed slightly from the previous results. These analyses showed that the pooled results for the primary outcome measures were robust both before and after the removal of relevant trials (**Tables 4**, **5**).

**Table 4:** Sensitivity analysis of objective response rate (ORR).

Types	Excluded trials (references)	Remaining trials	Statistical methods	Pooled RRs (95% CI)	P	I^2^
Participants number	108	32	REM	1.34 [1.24, 1.44]	<0.00001	0%
Treatment duration of CHIs	109	31	REM	1.35 [1.24, 1.47]	<0.00001	0%
Publication year	71	69	REM	1.39 [1.32, 1.46]	<0.00001	0%

REM, random-effects model; CI, confidence interval; RRs, risk ratios; CHIs, Chinese herbal injections.

**Table 5:** Sensitivity analysis of disease control rate (DCR).

Types	Excluded trials (references)	Remaining trials	Statistical methods	Pooled RRs (95% CI)	P	I^2^
Participants number	106	32	REM	1.18 [1.13, 1.24]	<0.00001	46%
Treatment duration of CHIs	107	31	REM	1.18 [1.11, 1.25]	<0.00001	52%
Publication year	70	68	REM	1.18 [1.15, 1.21]	<0.00001	20%

REM, random-effects model; CI, confidence interval; RRs, risk ratios; CHIs, Chinese herbal injections.

**Figure 8:**
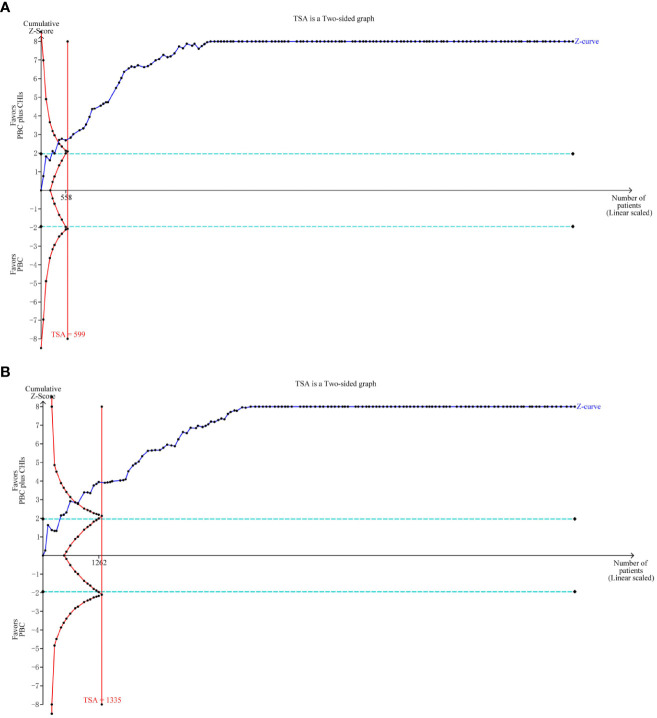
Trial sequential analysis (TSA) on outcomes. TSA analysis of ORR and DCR showed that the cumulative Z value had passed the traditional threshold value and TSA value, and the cumulative information had reached the expected value. **(A)** objective response rate; **(B)** disease control rate.

The required sample size of the TSA can estimate the meta-analysis and reduce the possibility of a false positive result. This study selected bilateral inspection method, setting I error probability for α at 0.05, statistical test force at 80%, merge relative risk loss at 20%, the incidence of positive events ORR control group at 35.83%, and the positive event rate in the DCR control group at 69.71%. TSA analysis of ORR and DCR showed that the cumulative Z value had passed the traditional threshold value and TSA value, and the cumulative information had reached the expected value. Therefore, TSA analysis confirmed that there was definite evidence that CHIs combined with PBC in the treatment of NSCLC could improve ORR and DCR (**Figure 8**).

## 4 Discussion

This study differs from the previous meta-analyses of CHIs and has certain advantages. First, there exists a wide variety of CHIs, and the efficacy of multiple CHIs when combined with PBC remains insufficiently compared. Therefore, we selected a variety of CHIs commonly used in clinical practice to conduct a systematic review of effectiveness and safety to provide a reference for the selection of CHIs in the treatment of NSCLC. Second, this study included high-quality literature and conducted detailed subgroup analysis and sensitivity tests according to different PBC chemotherapy regimens, yielding robust results.

Based on published RCTs, we conducted a meta-analysis of the efficacy and safety of CHIs when combined with PBC in the treatment of advanced NSCLC. Overall, compared with PBC, CHIs when combined with PBC exhibited a significant improvement in ORR, DCR, and QoL. And the incidence of leukopenia, anemia, thrombocytopenia, nausea and vomiting, diarrhea, constipation, and other adverse reactions was significantly reduced. Among them, the heterogeneity of ORR, DCR, QoL, diarrhea, and constipation was low, and the reliability of the results was high. Although the heterogeneity of leukopenia, anemia, thrombocytopenia, and nausea and vomiting indexes was high after the combined analysis, the sensitivity analysis showed that heterogeneity did not significantly affect the results and also had certain reliability. Sensitivity analysis showed that the results of the primary outcome index were also stable. In terms of methodological assessment, the overall quality of the included studies was moderate, and the GRADE assessment showed that the quality of evidence was low, which may be related to study design, publication bias, and heterogeneity.

PBC, as the first-line treatment for non-positive gene-driven advanced NSCLC, has been widely used in clinical practice and has a significant effect. However, the toxic side effects of PBC can interrupt the treatment process and reduce patients’ QoL Therefore, reducing adverse reactions while pursuing efficacy has become one of the urgent challenges in the treatment of NSCLC. TCM, which has been inherited for thousands of years, has accumulated rich experience in tumor treatment, which can be used as one of the ideas to solve the problem. Compound Kushen injection is an herbal extract of *Sophora flavescens* and *Smilax glabra*. Pharmacological studies have found that it can improve the immune level of patients, inhibit the proliferation of tumor cells, and play a role in anti-cancer therapy (164). Aidi injection is composed of extracts from cantharis, ginseng, *Astragalus membranaceus*, *Acanthopanax senticosusm*, and other drugs, with the main active ingredients being cantharidin, ginsenoside, and astragaloside. Many studies have shown that Aidi injection has anti-tumor activity, immunomodulatory effect, and adverse event reduction effect (12). The main components of Huachansu injection include toad venom ligands, alkaloids, and peptides. Pharmacological studies have shown that Huachansu injection has multiple anti-tumor effects, such as inducing tumor cell apoptosis, inhibiting tumor cell proliferation, invasion, and metastasis, and reversing drug resistance (165, 166). Shenmai, Shenfu, and Kangai injections all contain ginseng. Ginsenoside, the main component of ginseng, can induce lung cancer cell apoptosis and inhibit lung cancer cell proliferation (167, 168). However, the main components of Shenfu injection are ginsenoside compounds, which also have similar effects (169). In addition to ginseng, Shenmai injection also includes *Ophiopogon japonicus*, which has been shown to enhance cisplatin-induced apoptosis by regulating mitofusin-2 (Mfn2)-dependent mitochondrial dynamics in lung adenocarcinoma cells (170). Kangai injection can inhibit the proliferation of gastric cancer cells through IL-6/STAT3 pathway (171). Shenqifuzheng injection, which is composed of *Codonopsis pilosula* and *Astragalus membranaceus* extracts, has the effect of supplementing Qi deficiency and strengthening vital energy. Similar to the Shenmai injection, it has a synergistic effect on chemotherapy through cell cycle arrest and initiation of mitochondrial apoptosis, which is involved in the up-regulation of Mfn2 expression (172). The Kanglaite injection is another TCM injection extracted from coix seeds. Studies have found that its anti-tumor effect is related to inducing cancer cell apoptosis and improving immune function (173). The main component of Xiaoaiping injection is marsdenia tenacissima. Modern pharmacological studies have confirmed that the clearance vine can induce apoptosis and inhibit autophagy in NSCLC cells to play an anti-tumor role (174).

From the perspective of clinical practice, our results also show that CHIs based on Chinese herbal extracts can improve the efficacy of PBC in NSCLC and reduce the incidence of adverse reactions. Although the results showed that CHIs when combined with PBC were more effective and safer than PBC alone in the treatment of advanced NSCLC, it was unclear which CHIs played a role in this process; therefore, we conducted a subgroup analysis according to the type of CHIs.

Regarding the primary outcome indicators, ORR and DCR, 9 CHIs including Aidi injection, Compound Kushen injection, Huachansu injection, Kanglaite injection, Kangai injection, Lanxiangxi injection, Shenmai injection, Shenqifuzheng injection, and Xiaoaiping injection could improve ORR and DCR. Additionally, the heterogeneity of the combined analysis of 7 CHIs such as Aidi injection, Compound Kushen injection, Kanglaite injection, Kangai injection, Shenmai injection, Shenqifuzheng injection, and Xiaoaiping injection was low, and the reliability of the results was high. The heterogeneity of Huachansu injection in DCR and Lanxiangxi injection in ORR and DCR was high; however, because heterogeneity did not affect the results, the combined results of the two injections also have certain reliability. It is worth noting that Shenfu injection exhibited particularly unique effects, playing a positive role in DCR improvement; however, its effect on ORR improvement was not significant. The two CHIs of Javanica oil emulsion injection and Lentinan injection had no significant effect on improving ORR and DCR, and the heterogeneity of Javanica oil emulsion injection was low, yielding highly reliable results. However, Shenfu injection and Lentinan injection were not combined owing to the small number of studies, and the reliability of the results was hence relatively insufficient.

In terms of QoL improvement, 9 CHIs, Aidi injection, Compound Kushen injection, Huachansu injection, Javanica oil emulsion injection, Kanglaite injection, Kangai injection, Shenmai injection, Shenqifuzheng injection, and Xiaoaiping injection could improve QoL. The heterogeneity of the above injections was low, and the reliability of the results was high. Lanxiangxi injection had no obvious advantage; however, due to the small number of studies, The studies on Lanxiangxi injection was not combined. Hence, the results need to be further evaluated for reliability.

Regarding safety improvement, Aidi injection, Compound Kushen injection, Shenmai injection, Shenqifuzheng injection, Xiaoaiping injection and other five types of CHIs played a positive role in bone marrow suppression. Compound Kushen injection, Shenqifuzheng injection, Xiaoaiping injection, and the other three CHIs exhibited low heterogeneity and high reliability after combination. In contrast, Aidi injection and Shenmai injection had high heterogeneity in terms of the incidence of leukopenia and thrombocytopenia after combination. However, the heterogeneity did not affect the results; hence, the effects of the combined injection also have certain reliability. At the same time, studies have shown that Lanxiangxi injection has no apparent advantage in the prevention and treatment of bone marrow suppression, and the heterogeneity of leukopenia and thrombocytopenia after the combination is high. However, the heterogeneity does not affect the results. Therefore, the combined effects of this injection also have some reliability. Among other injections, Kangai injection and Shenfu injection played a positive role in leukopenia and thrombocytopenia; however, owing to the small number of literature, the reliability of the results needs to be further verified. Kangai injection showed no apparent advantage in alleviating the incidence of anemia. At the same time, Huachansu injection played a positive role in Leukopenia. However, these interpretations excluded the results after the combination of studies with higher heterogeneity, which needs to be further confirmed. Javanica oil emulsion injection did not show obvious advantages. It is worth noting that Kanglaite injection did not offer apparent advantages in alleviating anemia and thrombocytopenia incidence, and the heterogeneity in Anemia was high. However, the combined results were also reliable, because they did not affect the results. It showed an advantage in alleviating the incidence of leukopenia, but it needs to be further confirmed because it is the result of excluding the pooled studies with high heterogeneity. Compound Kushen injection had a positive effect on the digestive tract reaction. In terms of other injections such as Kanglaite injection, Kangai injection, Shenfu injection, and Shenmai injection, six types of CHIs such as Shenqifuzheng injection played a positive role in alleviating the incidence of nausea and vomiting. Among them, Kangai injection and Shenqifuzheng injection had high heterogeneity after being combined. However, the heterogeneity did not affect the results; hence, the combined results of these two injections also have certain reliability. However, the Shenfu injection was not combined due to the small number of studies, and the reliability of the results needs to be further verified. Javanica oil emulsion injection did not show any advantage in alleviating the incidence of nausea, vomiting, and diarrhea. However, owing to the poor availability of studies, the reliability of the results still needs to be further verified. Xiaoaiping injection showed an advantage in nausea and vomiting but not in diarrhea. Aidi injection and Lanxiangxi injection did not show obvious advantages in nausea and vomiting. However, Aidi injection was the result of the combination of studies excluding high heterogeneity; therefore, it needs to be further confirmed. The heterogeneity of Lanxiangxi injection was higher, but the heterogeneity did not affect the results.

In summary, the Compound Kushen injection has shown positive effects in improving the efficacy and QoL and reducing side effects, which can be recommended for clinical application. Aidi injection, Kanglaite injection, Kangai injection, Shenmai injection, Shenqifuzheng injection, and CHIs such as Xiaoaiping injection positively improve the efficacy and QoL. However, they exhibit insufficient effects in reducing toxic and side effects and hence can be used for secondary recommendations. Finally, Huachansu injection, Lanxiangxi injection, Javanica oil emulsion injection, Lentinan injection, Shenfu injection and other CHIs only showed advantages in some indicators, indicating that CHIs may not be recommended for the treatment of advanced NSCLC when the evidence is insufficient, and the research on these CHIs should be strengthened in the future for more reliable evidence-based medical application.

This study also has some limitations. First, all the included studies were conducted in China, which may have caused regional bias. Second, the number of studies on some CHIs is small, and hence, the efficacy may be exaggerated; therefore, the results obtained need to be further verified. Third, the study’s results showed asymmetric funnel plots and possible publication bias. Fourth, most included studies did not mention allocation concealment and blinding. Although our study included the RCTs with the highest level of evidence, methodologic shortcomings may exaggerate the clinical effect and further affect the credibility of the results. Fifth, the study period of the included studies was short, and the long-term endpoints such as OS and PFS were not reported; therefore, the level of evidence is insufficient when applied to the long-term treatment of tumors. In general, the number of studies on some CHIs is limited, and the overall quality of research needs further improvement. In the future, high-quality RCTs with long-term endpoint results are needed to further verify the efficacy and safety of CHI in combination with PBC in advanced NSCLC treatment.

## 5 Conclusion

From the available evidence, CHIs combined with PBC in the treatment of advanced NSCLC can improve ORR, DCR and QoL and alleviate the occurrence of adverse reactions such as leukopenia, anemia, thrombocytopenia, nausea and vomiting, diarrhea, and constipation. Among the studied CHIs, Compound Kushen injection has advantages in efficacy and safety and can be recommended for clinical application. However, considering the limitations of the existing evidence, more high-quality RCTs are needed to further verify the application of CHIs. Moreover, clinicians are advised to be cautious when applying the results of this study.

## Data availability statement

The original contributions presented in the study are included in the article/**Supplementary Material**. Further inquiries can be directed to the corresponding author.

## Author contributions

KC: Conceptualization, Data curation, Formal analysis, Investigation, Methodology, Project administration, Software, Visualization, Writing – original draft, Writing – review & editing. SH: Conceptualization, Data curation, Formal analysis, Software, Visualization, Writing – original draft, Writing – review & editing. DW: Conceptualization, Data curation, Formal analysis, Software, Visualization, Writing – original draft, Writing – review & editing. CQ: Data curation, Writing – original draft. ZW: Data curation, Writing – original draft. JW: Data curation, Writing – original draft. WH: Conceptualization, Project administration, Resources, Supervision, Writing – review & editing.

## Supplementary material

Supplementary Figure 1 Risk of bias summary. Methodological quality evaluation of the included literature.

Supplementary Figure 2 Forest plot of leukopenia in PBC versus PBC plus CHIs. The meta-analysis results, stratified by the type of CHIs, showed differences in leukopenia between PBC and PBC plus CHIs. CHIs, Chinese herbal injections; PBC, platinum-based chemotherapy.

Supplementary Figure 3 Forest plot of anemia in PBC versus PBC plus CHIs. The meta-analysis results, stratified by the type of CHIs, showed differences in anemia between PBC and PBC plus CHIs. CHIs, Chinese herbal injections; PBC, platinum-based chemotherapy.

Supplementary Figure 4 Forest plot of thrombocytopenia in PBC versus PBC plus CHIs. The meta-analysis results, stratified by the type of CHIs, showed differences in thrombocytopenia between PBC and PBC plus CHIs. CHIs, Chinese herbal injections; PBC, platinum-based chemotherapy.

Supplementary Figure 5 Forest plot of nausea and vomiting in PBC versus PBC plus CHIs. The meta-analysis results, stratified by the type of CHIs, showed differences in nausea and vomiting between PBC and PBC plus CHIs. CHIs, Chinese herbal injections; PBC, platinum-based chemotherapy.

Supplementary Figure 6 Forest plot of diarrhea in PBC versus PBC plus CHIs. The meta-analysis results, stratified by the type of CHIs, showed differences in diarrhea between PBC and PBC plus CHIs. CHIs, Chinese herbal injections; PBC, platinum-based chemotherapy.

Supplementary Figure 7 Forest plot of constipation in PBC versus PBC plus CHIs. The meta-analysis results, stratified by the type of CHIs, showed differences in constipation between PBC and PBC plus CHIs. CHIs, Chinese herbal injections; PBC, platinum-based chemotherapy.
