# Supplementary material for: Clinical efficacy and safety of Chinese herbal injections in combination with platinum-based chemotherapy for advanced non-small cell lung cancer: a systematic review and meta-analysis of 140 randomized controlled trials
Source: Front Oncol. 2024 Feb 2;14:1307836. doi: 10.3389/fonc.2024.1307836 (PMC10869539; doi:10.3389/fonc.2024.1307836)
Supplement: Supplementary Figure 1 — Risk of bias summary. Methodological quality evaluation of the included literature. [file DataSheet_1.docx]

Supplementary Material

**Clinical Efficacy and Safety of Chinese Herbal Injections in Combination with Platinum-Based Chemotherapy for Advanced Non-Small Cell Lung Cancer: A Systematic Review and Meta-analysis of 140 Randomized Controlled Trials**

Kangdi Cao, Shuaihang Hu, Dandan Wang, Chenxi Qiao, Zhuo Wang, Jinkun Wang, Wei Hou ^*^

*** Correspondence:** Wei Hou: [houwei1964@163.com](mailto:houwei1964@163.com)

# File 1: All databases’ search strategy

**1.1 Searching words of Chinese herbal injections**

| Names of Chinese Herbal injections | English Searching Words | Chinese Searching Words |
| --- | --- | --- |
| 中药注射剂 | Chinese herbal injection OR Chinese medicine injection OR injection of TCM | 中药注射剂 OR 中药注射液 |
| 艾迪注射液 | Aidi | 艾迪注射液OR艾迪注射剂OR爱迪注射液OR爱迪注射剂OR注射用艾迪OR艾迪液 |
| 蟾酥注射液 | Chansu OR Toad venom | 佳素OR史君轻OR蟾毒康OR蟾酥注射液OR蟾酥注射剂 |
| 复方苦参注射液 | Compound matrine OR Compound Kushen OR Fufangkushen | 岩舒注射液OR岩舒 OR 复方苦参注射液OR 复方苦参注射剂OR 复方苦参 |
| 华蟾素注射液 | Huachansu OR Cinobufacini | 华蟾素注射液 OR 华蟾素注射剂OR华蟾素 |
| 康莱特注射液 | Kanglaite | ZCE-3静脉乳OR薏苡仁提取液OR注射薏苡仁油OR薏苡仁酯OR康莱特注射液OR康莱特注射剂 |
| 消癌平注射液 | Xiaoaiping OR Xiao-Ai-Ping OR Marsdenia Tenacissima | 通关藤提取物OR通关藤注射液OR消癌平注射液OR消癌平注射剂OR消癌平 |
| 鸦胆子油乳注射液 | Yadanziyouru OR Javanica oil emulsion OR Bmcea javanica | 安体康注射液OR鸦胆子油乳注射液OR鸦胆子油乳注射剂OR鸦胆子油乳 |
| 参芪扶正注射液 | Shenqifuzheng | 参芪扶正注射液OR参芪扶正注射剂OR参芪扶正 |
| 康艾注射液 | Kangai | 康艾液OR康艾注射液OR康艾注射剂 |
| 参附注射液 | Shenfu | 参附注射液 OR 参附注射剂 OR 注射用参附 |
| 黄芪注射液 | Huangqi OR Astragalus | 黄芪注射液OR黄芪注射剂 OR 注射用黄芪冻干粉 |
| 香菇多糖注射液 | Xiangguduotang OR Lentinan | 力提能 OR 天地欣 OR香菇多糖注射液OR香菇多糖注射剂OR注射用香菇多糖 |
| 参麦注射液 | Shenmai | 参麦注射液OR参麦注射剂OR注射用参麦 |
| 榄香烯注射液 | Lanxiangxi OR Elemene | 榄香烯注射液 OR 榄香注射液 OR β-榄香烯注射液 OR 榄香烯脂质体注射液 OR 榄香烯乳注射液 |

**1.2 Searching words of Carcinoma, Non-Small-Cell Lung**

| Names of Carcinoma, Non-Small-Cell Lung | English Searching Words | Chinese Searching Words |
| --- | --- | --- |
| 非小细胞肺癌 | Carcinoma, Non-Small-Cell Lung OR Carcinoma, Non Small Cell Lung OR Carcinomas, Non-Small-Cell Lung OR Lung Carcinoma, Non-Small-Cell OR Lung Carcinomas, Non-Small-Cell OR Non-Small-Cell Lung Carcinomas OR Non-Small-Cell Lung Carcinoma OR Non Small Cell Lung Carcinoma OR Carcinoma, Non-Small Cell Lung OR Non-Small Cell Lung Carcinoma OR Non-Small Cell Lung Cancer OR Nonsmall Cell Lung Cancer OR NSCLC | 非小细胞肺癌 OR 肺癌 OR 肺部肿物 OR 肺部肿瘤OR SU= NSCLC |

**1.3 Search strategy of CNKI**

((SU=中药注射剂 OR SU=中药注射液 OR SU=艾迪注射液 OR SU=艾迪注射剂 OR SU=爱迪注射液 OR SU=爱迪注射剂 OR SU=注射用艾迪 OR SU=艾迪液 OR SU=佳素 OR SU=史君轻 OR SU=蟾毒康 OR SU=蟾酥注射液 OR SU=蟾酥注射剂 OR SU=岩舒注射液 OR SU=岩舒 OR SU=复方苦参注射液 OR SU=复方苦参注射剂 OR SU=复方苦参 OR SU=华蟾素注射液 OR SU=华蟾素注射剂 OR SU=华蟾素 OR SU=ZCE-3静脉乳 OR SU=薏苡仁提取液 OR SU=注射薏苡仁油 OR SU=薏苡仁酯 OR SU=康莱特注射液 OR SU=康莱特注射剂 OR SU=通关藤提取物 OR SU=通关藤注射液 OR SU=消癌平注射液 OR SU=消癌平注射剂 OR SU=消癌平 OR SU=安体康注射液 OR SU=鸦胆子油乳注射液 OR SU=鸦胆子油乳注射剂 OR SU=鸦胆子油乳 OR SU=参芪扶正注射液 OR SU=参芪扶正注射剂 OR SU=参芪扶正 OR SU=康艾液 OR SU=康艾注射液 OR SU=康艾注射剂 OR SU=参附注射液 OR SU=参附注射剂 OR SU=注射用参附 OR SU=黄芪注射液 OR SU=黄芪注射剂 OR SU=注射用黄芪冻干粉 OR SU=力提能 OR SU=天地欣 OR SU=香菇多糖注射液 OR SU=香菇多糖注射剂 OR SU=注射用香菇多糖 OR SU=参麦注射液 OR SU=参麦注射剂 OR SU=注射用参麦 OR SU=榄香烯注射液 OR SU=榄香注射液 OR SU=β-榄香烯注射液 OR SU=榄香烯脂质体注射液 OR SU=榄香烯乳注射液) AND (SU=非小细胞肺癌 OR SU=肺癌 OR SU=肺部肿物 OR SU=肺部肿瘤 OR SU=NSCLC)) NOT (TI=鼠 OR TI=兔)

**1.4 Search strategy of VIP**

((M=中药注射剂 OR M=中药注射液 OR M=艾迪注射液 OR M=艾迪注射剂 OR M=爱迪注射液 OR M=爱迪注射剂 OR M=注射用艾迪 OR M=艾迪液 OR M=佳素 OR M=史君轻 OR M=蟾毒康 OR M=蟾酥注射液 OR M=蟾酥注射剂 OR M=岩舒注射液 OR M=岩舒 OR M=复方苦参注射液 OR M=复方苦参注射剂 OR M=复方苦参 OR M=华蟾素注射液 OR M=华蟾素注射剂 OR M=华蟾素 OR M=ZCE-3静脉乳 OR M=薏苡仁提取液 OR M=注射薏苡仁油 OR M=薏苡仁酯 OR M=康莱特注射液 OR M=康莱特注射剂 OR M=通关藤提取物 OR M=通关藤注射液 OR M=消癌平注射液 OR M=消癌平注射剂 OR M=消癌平 OR M=安体康注射液 OR M=鸦胆子油乳注射液 OR M=鸦胆子油乳注射剂 OR M=鸦胆子油乳 OR M=参芪扶正注射液 OR M=参芪扶正注射剂 OR M=参芪扶正 OR M=康艾液 OR M=康艾注射液 OR M=康艾注射剂 OR M=参附注射液 OR M=参附注射剂 OR M=注射用参附 OR M=黄芪注射液 OR M=黄芪注射剂 OR M=注射用黄芪冻干粉 OR M=力提能 OR M=天地欣 OR M=香菇多糖注射液 OR M=香菇多糖注射剂 OR M=注射用香菇多糖 OR M=参麦注射液 OR M=参麦注射剂 OR M=注射用参麦 OR M=榄香烯注射液 OR M=榄香注射液 OR M=β-榄香烯注射液 OR M= 榄香烯脂质体注射液 OR M=榄香烯乳注射液) AND (M=非小细胞肺癌 OR M=肺癌 OR M=肺部肿物 OR M=肺部肿瘤 OR M=NSCLC)) NOT (T=鼠 OR T=兔)

**1.5 Search strategy of Wanfang data**

主题:(“中药注射剂”or “中药注射液”or “艾迪注射液”or “艾迪注射剂”or “爱迪注射液”or“爱迪注射剂”or“注射用艾迪”or“艾迪液”or“佳素”or“史君轻”or“蟾毒康”or“蟾酥注射液”or“蟾酥注射剂”or“岩舒注射液”or“岩舒”or“复方苦参注射液”or“复方苦参注射剂”or“复方苦参”or“华蟾素注射液”or“华蟾素注射剂”or“华蟾素”or“ZCE-3静脉乳”or“薏苡仁提取液”or“注射薏苡仁油”or“薏苡仁酯”or“康莱特注射液”or“康莱特注射剂”or“通关藤提取物”or“通关藤注射液”or“消癌平注射液”or“消癌平注射剂”or“消癌平”or“安体康注射液”or“鸦胆子油乳注射液”or“鸦胆子油乳注射剂”or“鸦胆子油乳”or“参芪扶正注射液”or“参芪扶正注射剂”or“参芪扶正”or“康艾液”or“康艾注射液”or“康艾注射剂”or“参附注射液”or“参附注射剂”or“注射用参附”or“黄芪注射液”or“黄芪注射剂”or“注射用黄芪冻干粉”or“力提能”or“香菇多糖注射液”or“香菇多糖”or“参麦”or“榄香烯”or“榄香烯注射液”or“β-榄香烯注射液”) and 主题:(“非小细胞肺癌”or“肺癌”or“肺部肿物”or“肺部肿瘤”or“NSCLC”) not (题名:“兔”or“鼠”)

**1.6 Search strategy of SinoMed**

((("非小细胞肺癌"[不加权:扩展] OR "NSCLC"[不加权:扩展]) OR "肺癌"[不加权:扩展] OR "肺部肿瘤"[不加权:扩展]) OR "肺部肿物"[不加权:扩展]) AND

("中药注射剂"[常用字段:智能] OR "中药注射液"[常用字段:智能] OR "艾迪注射液"[常用字段:智能] OR "艾迪注射剂"[常用字段:智能] OR "爱迪注射液"[常用字段:智能] OR "爱迪注射剂"[常用字段:智能] OR "注射用艾迪"[常用字段:智能] OR "艾迪液"[常用字段:智能] OR "佳素"[常用字段:智能] OR "史君轻"[常用字段:智能] OR "蟾毒康"[常用字段:智能] OR "蟾酥注射液"[常用字段:智能] OR "蟾酥注射剂"[常用字段:智能] OR "岩舒注射液"[常用字段:智能] OR "岩舒"[常用字段:智能] OR "复方苦参注射液"[常用字段:智能] OR "复方苦参注射剂"[常用字段:智能] OR "复方苦参"[常用字段:智能] OR "华蟾素注射液"[常用字段:智能] OR "华蟾素注射剂"[常用字段:智能] OR "华蟾素"[常用字段:智能] OR "ZCE-3静脉乳"[常用字段:智能] OR "薏苡仁提取液"[常用字段:智能] OR "注射薏苡仁油"[常用字段:智能] OR "薏苡仁酯"[常用字段:智能] OR "康莱特注射液"[常用字段:智能] OR "康莱特注射剂"[常用字段:智能] OR "通关藤提取物"[常用字段:智能] OR "通关藤注射液"[常用字段:智能] OR "消癌平注射液"[常用字段:智能] OR "消癌平注射剂"[常用字段:智能] OR "消癌平"[常用字段:智能] OR "安体康注射液"[常用字段:智能] OR "鸦胆子油乳注射液"[常用字段:智能] OR "鸦胆子油乳注射剂"[常用字段:智能] OR "鸦胆子油乳"[常用字段:智能] OR "参芪扶正注射液"[常用字段:智能] OR "参芪扶正注射剂"[常用字段:智能] OR "参芪扶正"[常用字段:智能] OR "康艾液"[常用字段:智能] OR "康艾注射液"[常用字段:智能] OR "康艾注射剂"[常用字段:智能] OR "参附注射液"[常用字段:智能] OR "参附注射剂"[常用字段:智能] OR "注射用参附"[常用字段:智能] OR "黄芪注射液"[常用字段:智能] OR "黄芪注射剂"[常用字段:智能] OR "注射用黄芪冻干粉"[常用字段:智能] OR "力提能"[常用字段:智能] OR "天地欣"[常用字段:智能] OR "香菇多糖注射液"[常用字段:智能] OR "香菇多糖注射剂"[常用字段:智能] OR "注射用香菇多糖"[常用字段:智能] OR "参麦注射液"[常用字段:智能] OR "参麦注射剂"[常用字段:智能] OR "注射用参麦"[常用字段:智能] OR "榄香烯注射液"[常用字段:智能] OR "榄香注射液"[常用字段:智能] OR "β-榄香烯注射液"[常用字段:智能] OR " 榄香烯脂质体注射液"[常用字段:智能] OR "榄香烯乳注射液)

**1.7 Search strategy of Pubmed**

| #1 | Carcinoma, Non-Small-Cell Lung [MeSH] OR Carcinoma, Non Small Cell Lung[tiab] OR Carcinomas, Non-Small-Cell Lung[tiab] OR Lung Carcinoma, Non-Small-Cell[tiab] OR Lung Carcinomas, Non-Small-Cell[tiab] OR Non-Small-Cell Lung Carcinomas[tiab] OR Non-Small-Cell Lung Carcinoma[tiab] OR Non Small Cell Lung Carcinoma[tiab] OR Carcinoma, Non-Small Cell Lung[tiab] OR Non-Small Cell Lung Carcinoma[tiab] OR Non-Small Cell Lung Cancer[tiab] OR Nonsmall Cell Lung Cancer[tiab] OR NSCLC[tiab] |
| --- | --- |
| #2 | (tumor*[tiab] OR carcinoma*[tiab] OR neoplasm*[tiab] OR cancer*[tiab]) AND (lung[tiab]) |
| #3 | #1 OR #2 |
| #4 | Chinese herbal injection*[tiab] OR Chinese medicine injection[tiab] OR injection of TCM[tiab] OR Shenqifuzheng[tiab] OR Kanglaite[tiab] OR Compound Kushen[tiab] OR Fufangkushen[tiab] OR Compound matrine[tiab] OR Aidi[tiab] OR Cinobufotalin injection[tiab] OR Huachansu[tiab] OR Xiaoaiping[tiab] OR Xiao-Ai-Ping[tiab] OR Marsdenia Tenacissima[tiab] OR Elemene[tiab] OR Lanxiangxi[tiab] OR Xiangguduotang[tiab] OR lentinan[tiab] OR javanica oil emulsion[tiab] OR Bmcea javanica[tiab] OR Yadanziyouru[tiab] OR kang'ai[tiab] OR kangai[tiab] OR kang-ai[tiab] OR Huangqi[tiab] OR Astragalus[tiab] OR Shenfu[tiab] OR Shenmai[tiab] |
| #5 | #3 AND #4 |

**1.8 Search strategy of Cochrane library**

#1 MeSH descriptor: [Carcinoma, Non-Small-Cell Lung] explode all trees

#2 ((tumor* OR carcinoma* OR neoplasm* OR cancer*) AND (lung)):ti,ab,kw

#3 #1 OR #2

#4 Chinese herbal injection*:ti,ab,kw or Chinese medicine injection:ti,ab,kw or injection of TCM:ti,ab,kw or Shenqifuzheng:ti,ab,kw or Kanglaite:ti,ab,kw or Compound Kushen:ti,ab,kw or Fufangkushen:ti,ab,kw or Compound matrine:ti,ab,kw or Aidi:ti,ab,kw or Cinobufotalin injection:ti,ab,kw or Huachansu:ti,ab,kw or Xiaoaiping:ti,ab,kw or Xiao-Ai-Ping:ti,ab,kw or Marsdenia Tenacissima:ti,ab,kw or Elemene:ti,ab,kw or Lanxiangxi:ti,ab,kw or Xiangguduotang:ti,ab,kw or lentinan:ti,ab,kw or javanica oil emulsion:ti,ab,kw or Bmcea javanica:ti,ab,kw or Yadanziyouru:ti,ab,kw or kang'ai:ti,ab,kw or kangai:ti,ab,kw or kang-ai:ti,ab,kw or Huangqi:ti,ab,kw or Astragalus:ti,ab,kw or Shenfu:ti,ab,kw or Shenmai:ti,ab,kw

#5 #3 AND #4

**1.9 Search strategy of Embase**

| #1 | 'non small cell lung cancer'/exp |
| --- | --- |
| #2 | (tumor*:ab,kw,ti OR carcinoma*:ab,kw,ti OR neoplasm*:ab,kw,ti OR cancer*:ab,kw,ti) AND (lung:ab,kw,ti) |
| #3 | #1 OR #2 |
| #4 | 'Chinese herbal injection*':ab,kw,ti OR 'Chinese medicine injection':ab,kw,ti OR 'injection of TCM':ab,kw,ti OR 'Shenqifuzheng':ab,kw,ti OR 'Kanglaite':ab,kw,ti OR 'Compound Kushen':ab,kw,ti OR 'Fufangkushen':ab,kw,ti OR 'Compound matrine':ab,kw,ti OR 'Aidi':ab,kw,ti OR 'Cinobufotalin injection':ab,kw,ti OR 'Huachansu':ab,kw,ti OR 'Xiaoaiping':ab,kw,ti OR 'Xiao-Ai-Ping':ab,kw,ti OR 'Marsdenia Tenacissima':ab,kw,ti OR 'Elemene':ab,kw,ti OR 'Lanxiangxi':ab,kw,ti OR 'Xiangguduotang':ab,kw,ti OR 'lentinan':ab,kw,ti OR 'javanica oil emulsion':ab,kw,ti OR 'Bmcea javanica':ab,kw,ti OR 'Yadanziyouru':ab,kw,ti OR 'kang'ai':ab,kw,ti OR 'kangai':ab,kw,ti OR 'kang-ai':ab,kw,ti OR 'Huangqi':ab,kw,ti OR 'Astragalus':ab,kw,ti OR 'Shenfu':ab,kw,ti OR 'Shenmai':ab,kw,ti |
| #5 | #3 AND #4 |

# Supplementary Figures and Tables

## Supplementary Figures


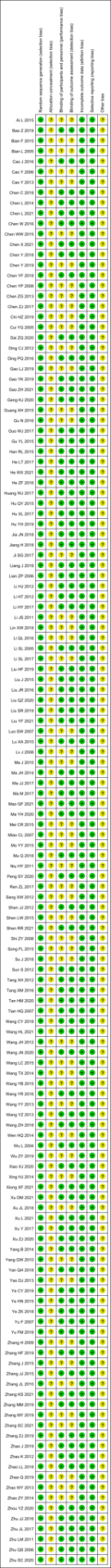


**Supplementary Figure 1. Risk of bias summary**


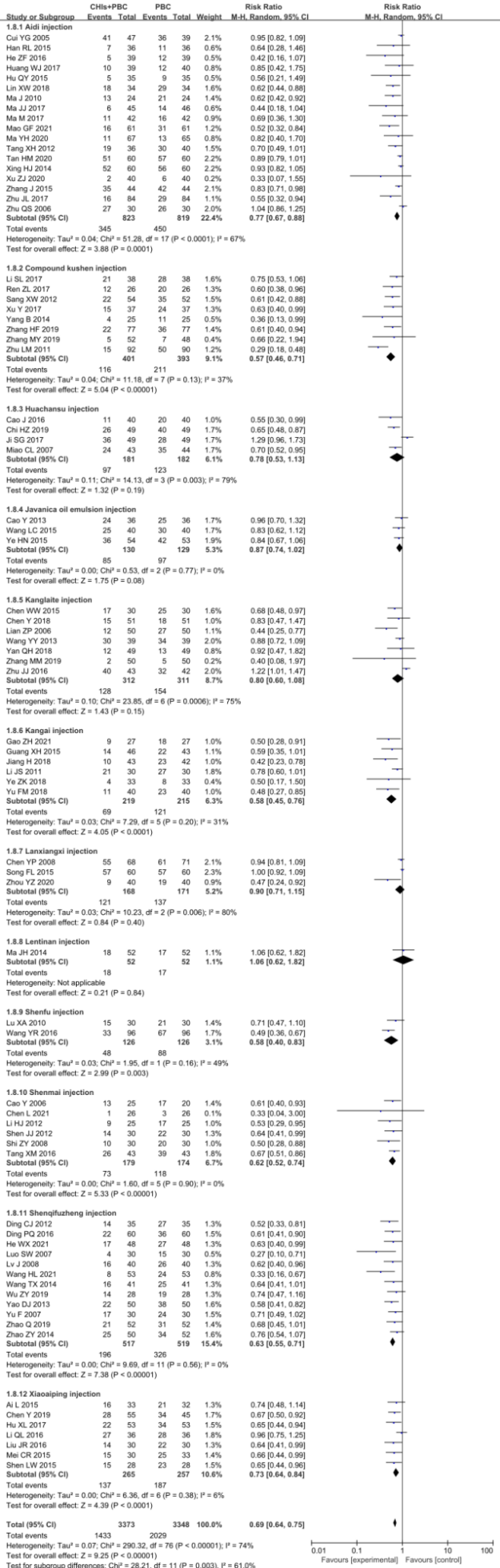


**Supplementary Figure 2.** **Forest plot of leukopenia in PBC versus PBC plus CHIs**


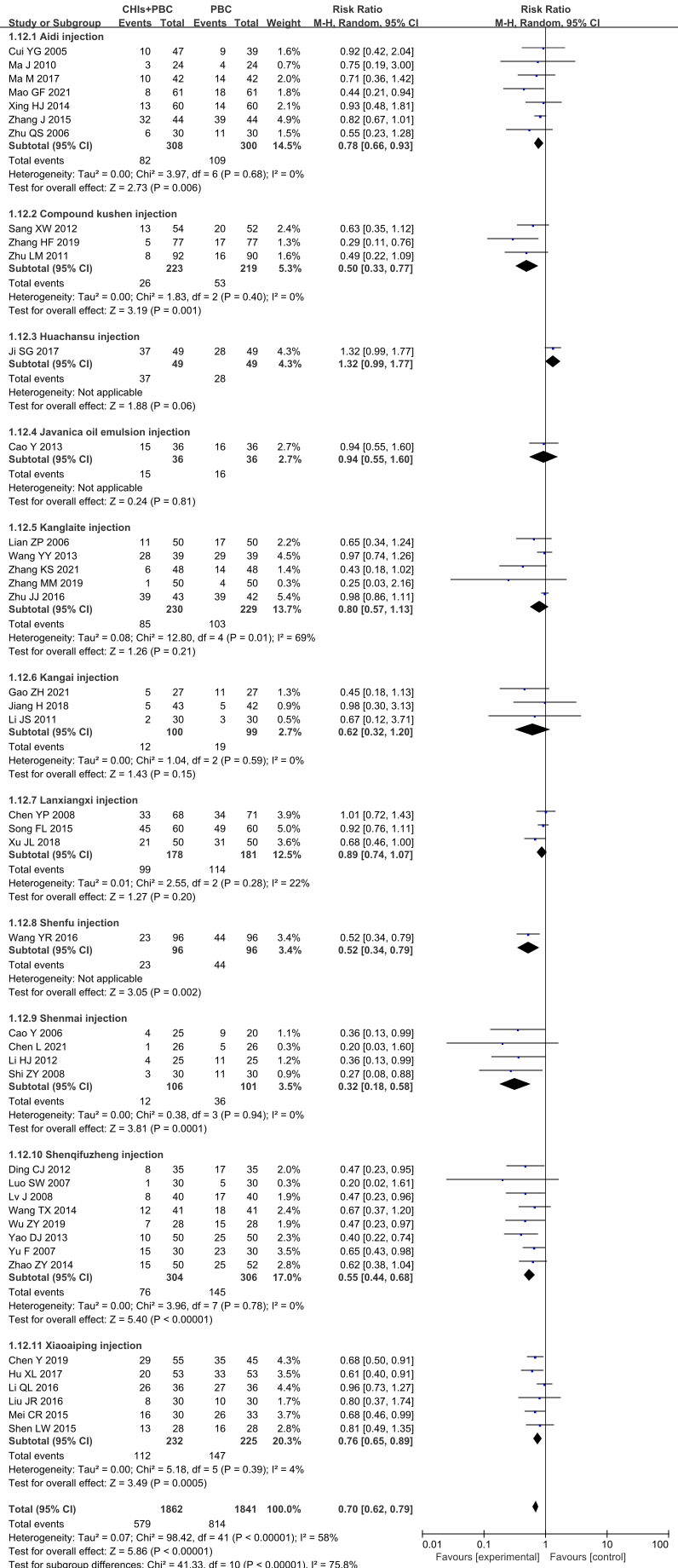


**Supplementary Figure 3. Forest plot of anemia in PBC versus PBC plus CHIs**


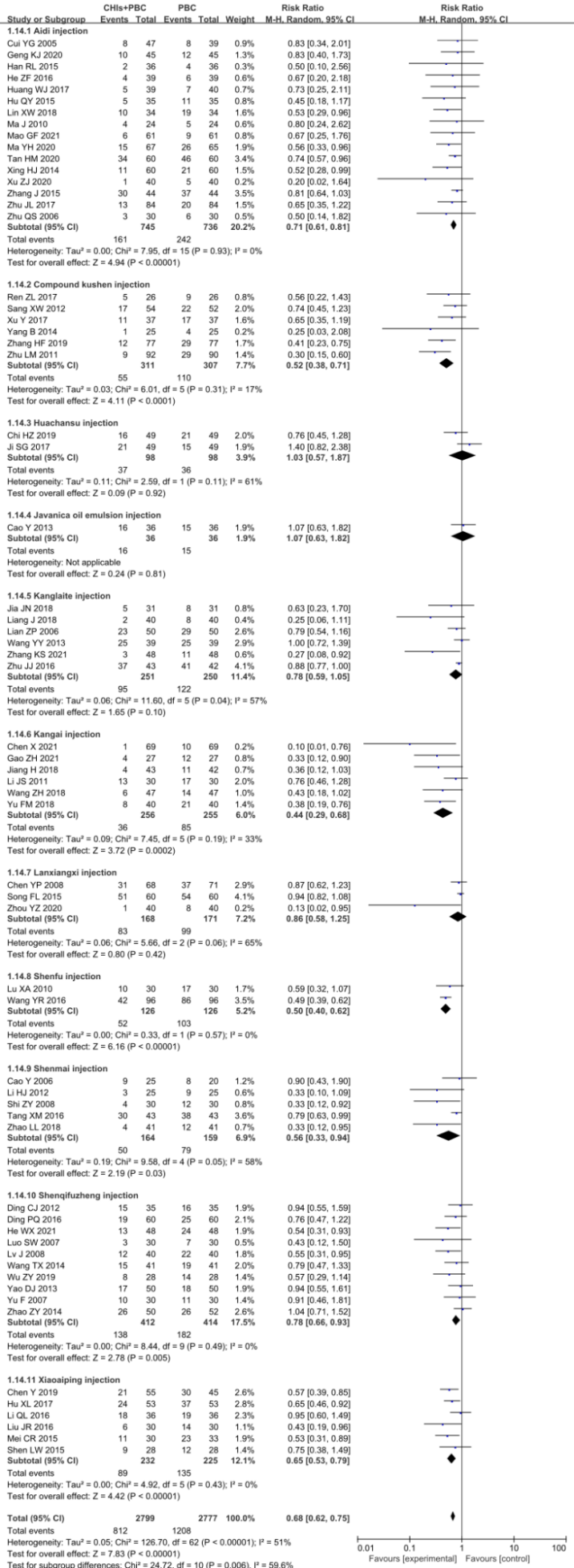


**Supplementary Figure 4. Forest plot of thrombocytopenia in PBC versus PBC plus CHIs**


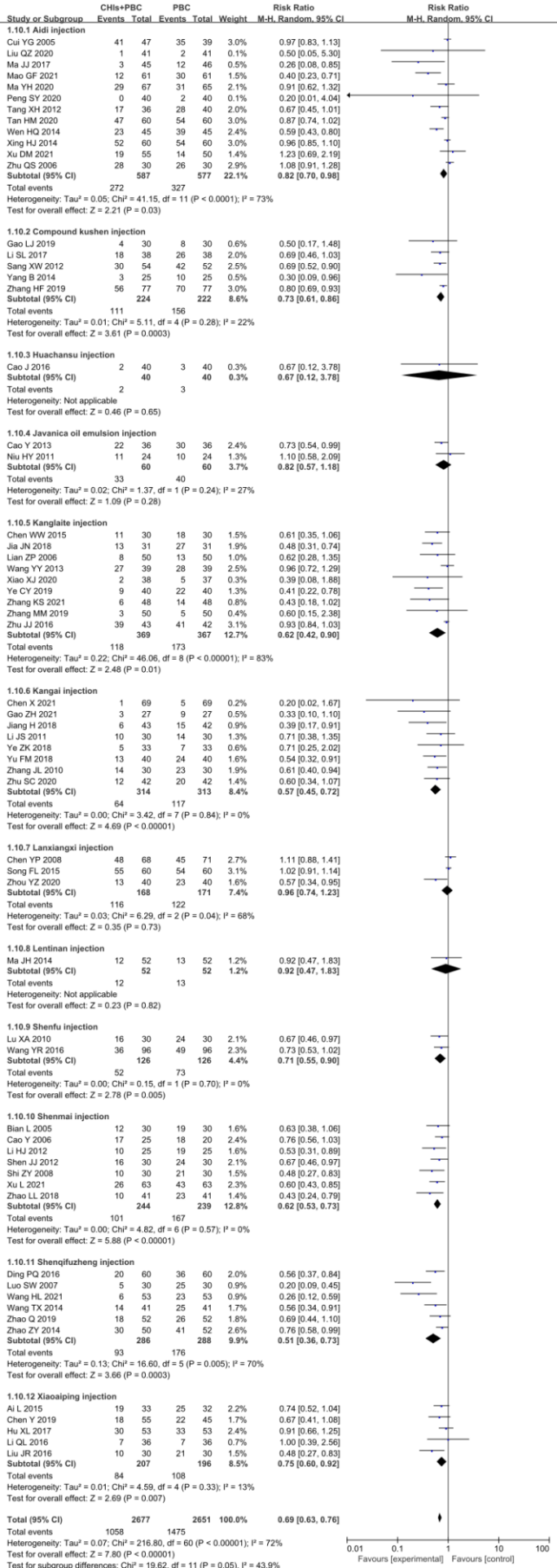


**Supplementary Figure 5. Forest plot of nausea and vomiting in PBC versus PBC plus CHIs**


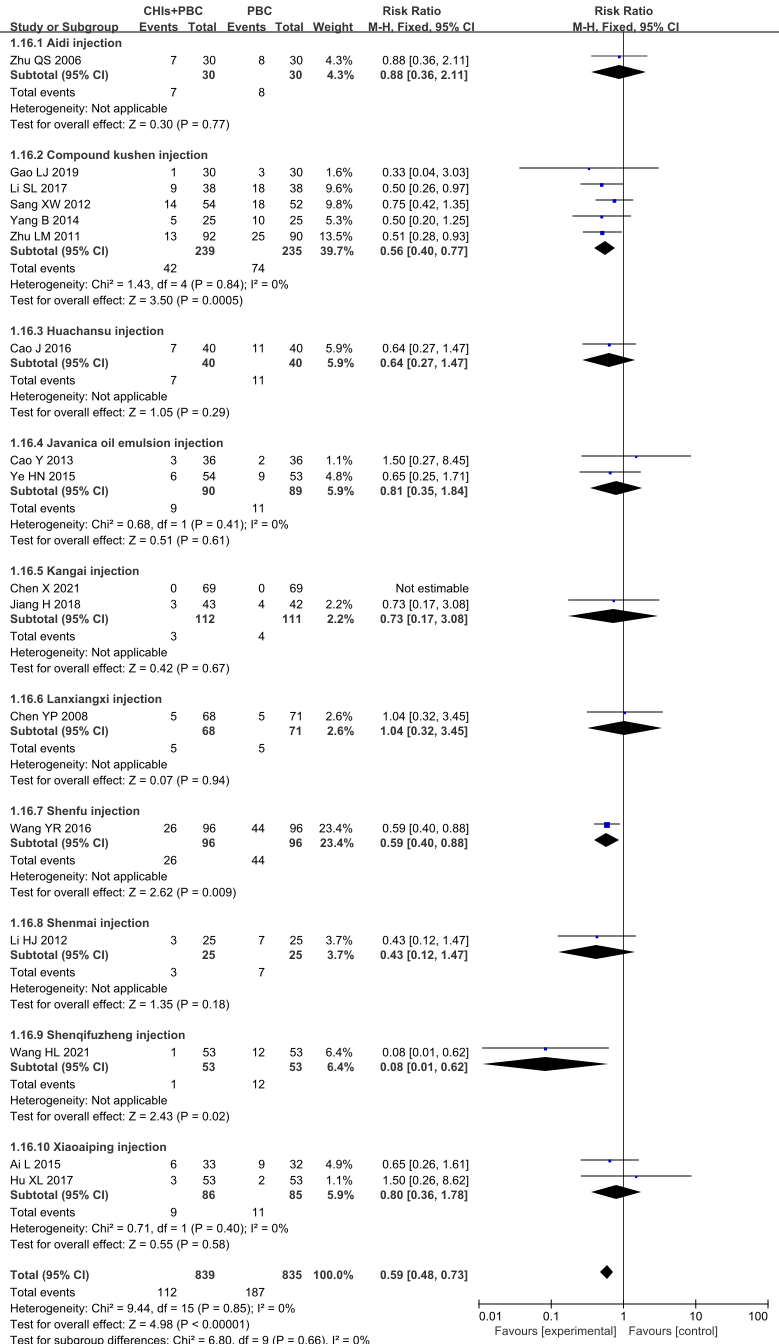


**Supplementary Figure 6. Forest plot of diarrhea in PBC versus PBC plus CHIs**


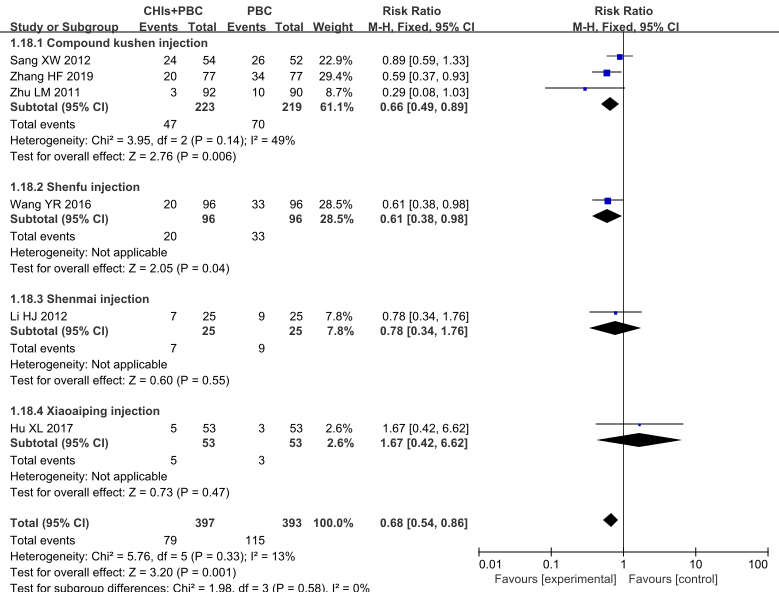


**Supplementary Figure 7. Forest plot of constipation in PBC versus PBC plus CHIs**

## Supplementary Table

**Supplementary Table 1. Basic characteristics of the Included Studies**

| Study ID | NSCLC（III—IV） | | | | Inventions | | | evaluation criteria of tumor response | evaluation criteria of adverse reactions | Outcome |
| --- | --- | --- | --- | --- | --- | --- | --- | --- | --- | --- |
|  | E/C | M/F | Age | Sq/Ad/Ot | Treatment | | Control |  |  |  |
|  |  |  |  |  | E | D/C |  |  |  |  |
| Ai L 2015 | 33/32 | 42/23 | 47-73 | 0/65/0 | XAP+DC | 60ml/d,  14 days/course,  2 courses | DC | Unclear | Unclear | ①②③④⑦⑧ |
| Bao Z 2019 | 47/47 | 61/33 | 65-71 | Unclear | SQFZ+GP | 250ml/d,  21 days/course,  3 courses | GP | Unclear | Unclear | ①② |
| Bian F 2015 | 32/31 | 33/30 | 25-56 | Unclear | HCS+GP | 20ml/d,  21 days/course,  4 courses | GP | RECIST | Unclear | ①②③ |
| Bian L 2005 | 30/30 | 45/15 | 37-71 | Unclear | SM+NP | 40-60ml/d,  14 days/course,  2 courses | NP | WHO | WHO | ①②③⑦ |
| Cao J 2016 | 40/40 | 49/31 | E: 57.41±9.04  C: 57.15±9.24 | 47/33/0 | HCS+DP | 20ml/d,  7 days/course,  4 courses | DP | RECIST | Unclear | ①②④⑦⑧ |
| Cao Y 2006 | 25/20 | 30/15 | 32-74 | 2/20/0 | SM+NP/GP | 40ml/d,  14 days/course,  3 courses | NP/GP | WHO | WHO | ①②③④⑤⑥⑦ |
| Cao Y 2013 | 36/36 | 40/32 | 40-73 | 32/39/1 | JOE+GP/DP | 30ml/d,  14 days/course,  2 courses | GP/DP | WHO | WHO | ①②③④⑤⑥⑦⑧ |
| Chen C 2018 | 30/30 | 31/29 | 35-63 | Unclear | KLT+GP | 200ml/d,  21 days/course,  1 courses | GP | RECIST | Unclear | ①② |
| Chen L 2014 | 41/42 | 53/30 | E: 62.4±10.5 C: 61.9±10.7 | 47/36/0 | KA+NP | 40ml/d,  21 days/course,  2 courses | NP | WHO | Unclear | ①② |
| Chen L 2021 | 26/26 | 38/14 | 30-71 | Unclear | SM+PC | 100ml/d,  14 days/course,  2 courses | PC | RECIST | Unclear | ①②④⑤ |
| Chen W 2016 | 44/44 | 47/41 | 55-78 | 20/58/10 | KLT+GP | Unclear,  30 days/course,  4 courses | GP | RECIST | Unclear | ①② |
| Chen WW 2015 | 30/30 | 34/26 | 36-71 | 20/40/0 | KLT+NP | 200ml/d,  21 days/course,  2 courses | NP | WHO | WHO | ①②③④⑦ |
| Chen X 2021 | 69/69 | 86/52 | 55-85 | 64/55/19 | KA+PP | 40ml/d,  10 days/course,  2 courses | PP | Unclear | Unclear | ①②⑥⑦⑧ |
| Chen Y 2018 | 51/51 | 59/43 | 57-79 | 28/58/16 | KLT+GP | 200ml/d,  21 days/course,  4 courses | GP | Unclear | Unclear | ①②④ |
| Chen Y 2019 | 55/45 | 66/34 | 32-68 | 34/63/3 | XAP+DC | 40ml/d,  15 days/course,  2 courses | DC | RECIST | Unclear | ①②③④⑤⑥⑦ |
| Chen YF 2018 | 40/40 | 45/35 | 42-77 | 61/19/0 | SQFZ+NP | 250ml/d,  14 days/course,  2 courses | NP | WHO | Unclear | ①② |
| Chen YP 2008 | 70/73 | 100/43 | 32-76 | Unclear | LXX+DP | 600mg/d,  14 days/course,  2 courses | DP | WHO | WHO | ①②③④⑤⑥⑦⑧ |
| Chen ZG 2013 | 54/52 | 72/34 | 57-78 | 31/21/54 | AD+DP | 50ml/d,  Unclear,  4 courses | DP | RECIST | Unclear | ①②③ |
| Chen ZJ 2017 | 31/30 | 31/30 | 40-80 | 0/31/30 | SQFZ+PP | 250ml/d,  21 days/course,  2 courses | PP | RECIST | WHO | ①②③ |
| Chi HZ 2019 | 49/49 | 54/44 | E: 55±10  C: 54±10 | 54/31/13 | HCS+DC | 10-20ml/d,  28 days/course,  3 courses | DC | RECIST | CTCAE | ①②③④⑥ |
| Cui YG 2005 | 47/39 | 51/35 | 34-78 | 41/45/0 | AD+NP | 50ml/d,  14 days/course,  2 courses | NP | WHO | WHO | ①②③④⑤⑥⑦ |
| Dai ZQ 2020 | 40/40 | 54/26 | 61-79 | Unclear | CKS+NP | 20ml/d,  12 days/course,  4 courses | NP | Unclear | Unclear | ①② |
| Ding CJ 2012 | 35/35 | 42/28 | 38-70 | 23/47/0 | SQFZ+GP/PP | 250ml/d,  10 days/course,  4 courses | GP/PP | WHO | WHO | ①②③④⑤⑥ |
| Ding PQ 2016 | 60/60 | 78/42 | 42-80 | 71/49/0 | SQFZ+NP | 250ml/d,  21 days/course,  2 courses | NP | WHO | Unclear | ①②④⑥⑦ |
| Gao LJ 2019 | 30/30 | 32/28 | 25-70 | 34/26/0 | CKS+GP | 20ml/d,  Unclear,  Unclear | GP | Unclear | CTCAE | ①②③⑦⑧ |
| Gao YK 2019 | 30/30 | 32/28 | 36-70 | 29/31/0 | KA+DP | 40ml/d,  21 days/course,  2 courses | DP | RECIST | NCI-CTC | ①② |
| Gao ZH 2021 | 27/27 | 33/21 | 37-76 | 16/29/9 | KA+GP | 40-60ml/d,  10 days/course,  4 courses | GP | RECIST | WHO | ①②④⑤⑥⑦ |
| Geng KJ 2020 | 45/45 | 61/29 | 44-79 | 50/31/9 | AD+GP | 50ml/d,  14 days/course,  4 courses | GP | Unclear | Unclear | ①②⑥ |
| Guang XH 2015 | 46/43 | 50/39 | 40-70 | 32/57/0 | KA+GP | 40ml/d,  14 days/course,  4 courses | GP | RECIST | WHO | ①②③④ |
| Gu N 2016 | 39/39 | 54/24 | 43-76 | Unclear | XAP+GP | 20ml/d,  21 days/course,  1 courses | GP | Unclear | Unclear | ①②③ |
| Guo WJ 2017 | 44/43 | 45/42 | 33-72 | 31/48/8 | AD+PC | 50ml/d,  21 days/course,  2 courses | PC | WHO | WHO | ①② |
| Gu YL 2015 | 39/39 | 53/25 | 34-72 | Unclear | CKS+DP | 20ml/d,  14 days/course,  4 courses | DP | WHO | Unclear | ①② |
| Han RL 2015 | 36/36 | 39/33 | 48-67 | Unclear | AD+GP | 50ml/d,  28 days/course,  3 courses | GP | WHO | Unclear | ①②④⑥ |
| He LT 2017 | 54/54 | 81/27 | 38-80 | 55/36/17 | KLT+DP | 100ml/d,  21 days/course,  3 courses | DP | Unclear | Unclear | ①② |
| He WX 2021 | 48/48 | 58/38 | 56-78 | 52/44/0 | SQFZ+GP | 250ml/d,  21 days/course,  4 courses | GP | WHO | Unclear | ①②④⑥ |
| He ZF 2016 | 39/39 | 27/51 | 46-70 | 16/62/0 | AD+DC | Unclear,  14 days/course,  Unclear | DC | RECIST | Unclear | ①②④⑥ |
| Huang WJ 2017 | 39/40 | 46/33 | 49-70 | 25/27/27 | AD+GP | 60ml/d,  21 days/course,  3 courses | GP | RECIST | WHO | ①②④⑥ |
| Hu QY 2015 | 35/35 | 41/29 | 34-76 | 31/28/11 | AD+DC | 50ml/d,  14 days/course,  2 courses | DC | WHO | Unclear | ①②④⑥ |
| Hu XL 2017 | 53/53 | 66/40 | 32-74 | 39/54/13 | XAP+GC | 20ml/d,  15 days/course,  4 courses | GC | WHO | WHO | ①②④⑤⑥⑦⑧⑨ |
| Hu YH 2019 | 39/39 | 51/27 | 50-75 | Unclear | XAP+PC | 60ml/d,  14 days/course,  4 courses | PC | WHO | Unclear | ①② |
| Jia JN 2018 | 31/31 | 34/28 | 43-74 | 27/31/4 | KLT+DP | 200mg/d,  15 days/course,  2 courses | DP | WHO | Unclear | ①②⑥⑦ |
| Jiang H 2018 | 43/42 | 52/33 | E: 58.85±10.16  C: 58.34±10.42 | 52/28/5 | KA+GP | 50ml/d,  14 days/course,  4 courses | GP | RECIST | CTCAE | ①②④⑤⑥⑦⑧ |
| Ji SG 2017 | 49/49 | 45/53 | 25-75 | 49/22/27 | KCS+DC | 20ml/d,  14 days/course,  4 courses | DC | RECIST | NCI-CTC | ①②③④⑤⑥ |
| Liang J 2018 | 40/40 | 44/36 | E: 54.69±4.94  C: 55.37±5.18 | 44/23/13 | KLT+NP | 10g/d,  21 days/course,  2 courses | NP | WHO | Unclear | ①②⑥ |
| Lian ZP 2006 | 50/50 | 67/33 | 31-71 | 52/32/16 | KLT+GP | 200ml/d,  10 days/course,  2 courses | GP | WHO | WHO | ①②④⑤⑥⑦ |
| Li HJ 2012 | 25/25 | 35/15 | 42-77 | 21/18/11 | SM+GP | 50ml/d,  10 days/course,  3 courses | GP | WHO | WHO | ①②④⑤⑥⑦⑧⑨ |
| Li HT 2012 | 30/30 | 44/16 | 49-82 | 19/41/0 | SQFZ+PP | 250ml/d,  10 days/course,  2 courses | PP | WHO | Unclear | ①②③ |
| Li HY 2017 | 41/41 | 43/39 | 55-75 | 56/18/8 | KLT+GP | 100ml/d,  21 days/course,  4 courses | GP | RECIST | Unclear | ①② |
| Li JS 2011 | 30/30 | 43/17 | 50-79 | 35/17/8 | KA+PP | 40ml/d,  14 days/course,  3 courses | PP | RECIST | WHO | ①②③④⑤⑥⑦ |
| Lin XW 2018 | 34/34 | 38/30 | E: 64.20±13.14  C: 59.67±11.55 | 29/39/0 | AD+PP | 50ml/d,  7 days/course,  4 courses | PP | WHO | Unclear | ①②③④⑥ |
| Li QL 2016 | 36/36 | 54/18 | 27-74 | 42/28/2 | XAP+GP | 40ml/d,  15 days/course,  2 courses | GP | RECIST | WHO | ①②③④⑤⑥⑦ |
| Li SL 2005 | 30/30 | 50/10 | 38-76 | 33/24/3 | SM+NP | 40-60ml/d,  21 days/course,  4-6 courses | NP | WHO | Unclear | ①② |
| Li SL 2017 | 38/38 | 49/27 | 35-72 | 37/33/6 | CKS+PP | 15ml/d,  14 days/course,  2 courses | PP | RECIST | Unclear | ①②③④⑦⑧ |
| Liu HF 2019 | 44/44 | 54/34 | 42-76 | 12/39/37 | AD+GP | 50ml/d,  21 days/course,  2 courses | GP | Unclear | Unclear | ①② |
| Liu J 2015 | 36/36 | 41/31 | 58-70 | 42/21/9 | LXX+GP | 600mg/d,  14 days/course,  3 courses | GP | Unclear | Unclear | ①② |
| Liu JR 2016 | 30/30 | 31/29 | 40-79 | 60/0/0 | XAP+GP | 40ml/d,  14 days/course,  2 courses | GP | RECIST | Unclear | ①②④⑤⑥⑦ |
| Liu QZ 2020 | 41/41 | 43/39 | 42-70 | 44/37/1 | AD+PP | 100ml/d,  10 days/course,  Unclear | PP | RECIST | Unclear | ①②⑦ |
| Liu SR 2019 | 49/49 | 51/47 | 45-88 | 34/34/30 | JOE+GP | 30ml/d,  21 days/course,  2 courses | GP | WHO | WHO | ①② |
| Liu YF 2021 | 34/34 | 52/16 | 53-77 | Unclear | SQFZ+GP | 250ml/d,  10 days/course,  2 courses | GP | Unclear | Unclear | ①② |
| Luo SW 2007 | 30/30 | 39/21 | 33-75 | 33/27/0 | SQFZ+PP | 250ml/d,  14 days/course,  2 courses | PP | WHO | WHO | ①③④⑤⑥⑦ |
| Lu XA 2010 | 30/30 | 43/17 | 38-70 | 18/36/6 | SF+NP | 60ml/d,  10 days/course,  2 courses | NP | WHO | Unclear | ①②④⑥⑦ |
| Lv J 2008 | 40/40 | 65/15 | 51-78 | 42/30/8 | SQFZ+NP | 250ml/d,  21 days/course,  2 courses | NP | WHO | WHO | ①②③④⑤⑥ |
| Ma J 2010 | 24/24 | 39/9 | E: 58.7  C: 59.1 | 17/19/12 | AD+GP | 40ml/d,  10 days/course,  2-4 courses | GP | WHO | WHO | ①②③④⑤⑥ |
| Ma JH 2014 | 52/52 | 54/50 | 33-73 | 42/35/27 | LE+NP | 1mg/d,  8 days/course,  2 courses | NP | Unclear | Unclear | ①②④⑦ |
| Ma JJ 2017 | 45/46 | 64/27 | E: 52.33±11.15  C: 51.54±11.62 | 0/91/0 | AD+NP | 60ml/d,  7 days/course,  6 courses | NP | RECIST | WHO | ①②④⑦ |
| Ma M 2017 | 42/42 | 55/29 | 44-75 | 9/44/31 | AD+GP | 50ml/d,  21 days/course,  4 courses | GP | RECIST | Unclear | ①②④⑤ |
| Mao GF 2021 | 61/61 | 86/36 | E: 49.81±7.17  C: 50.72±6.45 | 0/122/0 | AD+AP | 100ml/d,  10 days/course,  2-4 courses | AP | RECIST | WHO | ①②④⑤⑥⑦ |
| Ma YH 2020 | 67/65 | 72/61 | 44-74 | Unclear | AD+GC | 50ml/d,  28 days/course,  3 courses | GC | RECIST | Unclear | ①②④⑥⑦ |
| Mei CR 2015 | 30/33 | 38/25 | 37-79 | 34/19/10 | XAP+PP | 20ml/d,  21 days/course,  2 courses | PP | WHO | WHO | ①②③④⑤⑥ |
| Miao CL 2007 | 43/44 | 50/37 | 34-74 | 48/38/1 | HCS+NP | 20ml/d,  5 days/course,  3-6 courses | NP | WHO | WHO | ①②③④ |
| Mo YY 2015 | 43/43 | 49/37 | E: 56.96±6.17  C: 57.28±6.32 | 31/55/0 | AD+DP | 50ml/d,  14 days/course,  2 courses | DP | WHO | Unclear | ①②③ |
| Mu Q 2018 | 47/47 | 56/38 | 45-76 | Unclear | KLT+DC | 200mg/d,  20 days/course,  Unclear | DC | Unclear | Unclear | ①② |
| Niu HY 2011 | 24/24 | 37/11 | 35-75 | 25/23/0 | JOE+DP | 30ml/d,  14 days/course,  2 courses | DP | RECIST | WHO | ①②③⑦ |
| Peng SY 2020 | 40/40 | 43/37 | 42-75 | Unclear | AD+NP | 60-100ml/d,  10 days/course,  2 courses | NP | RECIST | Unclear | ①②⑦ |
| Ren ZL 2017 | 26/26 | 36/16 | 61-85 | Unclear | CKS+DP | 20ml/d,  14 days/course,  3 courses | DP | RECIST | WHO | ①②③④⑥ |
| Sang XW 2012 | 54/52 | 65/41 | 31-73 | 43/63/0 | CKS+NP | 20ml/d,  14 days/course,  2 courses | NP | WHO | WHO | ①②③④⑤⑥⑦⑧⑨ |
| Shen JJ 2012 | 30/30 | 50/10 | 43-72 | 15/45/0 | SM+NP | 60ml/d,  14 days/course,  2 courses | NP | WHO | WHO | ①②④⑦ |
| Shen LW 2015 | 28/28 | 39/17 | 50-75 | 23/33/0 | XAP+PP | 40-60ml/d,  10-15 days/course,  2 courses | PP | WHO | NCI-CTC | ①②③④⑤⑥ |
| Shen RR 2021 | 30/30 | 40/20 | 34-81 | 31/29/0 | AD+GP | 60ml/d,  21 days/course,  4 courses | GP | Unclear | Unclear | ①② |
| Shi ZY 2008 | 30/30 | 42/18 | 42-79 | 18/42/0 | SM+NP | 60ml/d,  15 days/course,  2 courses | NP | Unclear | WHO | ①②③④⑤⑥⑦ |
| Song FL 2015 | 60/60 | 72/48 | 30-78 | 42/69/9 | LXX+NP | 400mg/d,  8 days/course,  4 courses | NP | RECIST | WHO | ①②③④⑤⑥⑦ |
| Su J 2018 | 42/42 | 35/49 | 34-68 | 0/84/0 | KLT+AP | 200ml/d,  21 days/course,  2 courses | AP | WHO | WHO | ①②③ |
| Sun S 2012 | 35/35 | 41/29 | 35-75 | 38/22/10 | KLT+GP | 200ml/d,  20 days/course,  4 courses | GP | RECIST | Unclear | ①② |
| Tang XH 2012 | 36/40 | 42/34 | 38-73 | 23/45/8 | AD+DP | 50ml/d,  14 days/course,  2 courses | DP | RECIST | WHO | ①②④ |
| Tang XM 2016 | 43/43 | 52/34 | 34-72 | 0/86/0 | SM+GP | 50ml/d,  21 days/course,  3 courses | GP | RECIST | NCI-CTC | ①②④⑥⑦ |
| Tan HM 2020 | 60/60 | 78/42 | E: 67.24±5.92  C: 66.89±6.38 | 64/49/7 | AD+GP | 50ml/d,  10 days/course,  2 courses | GP | WHO | Unclear | ①②④⑥⑦ |
| Tian HQ 2007 | 58/57 | 79/36 | 42-78 | 43/51/21 | JOE+GP | 40ml/d,  14 days/course,  2 courses | GP | WHO | Unclear | ①② |
| Wang CY 2018 | 42/42 | 45/39 | 55-78 | 20/54/10 | KLT+DP | 100ml/d,  20 days/course,  4 courses | DP | RECIST | Unclear | ① |
| Wang HL 2021 | 53/53 | 58/48 | 47-73 | Unclear | SQFZ+GP | 250ml/d,  21 days/course,  2 courses | GP | Unclear | Unclear | ①②④⑦⑧ |
| Wang JH 2012 | 68/68 | 94/42 | 52-74 | 99/37/0 | JOE+GP | 30ml/d,  30 days/course,  4 courses | GP | WHO | WHO | ①②③ |
| Wang JN 2020 | 43/43 | 55/31 | E: 63.26±2.57  C: 63.02±2.34 | Unclear | CKS+GP | 20ml/d,  21 days/course,  2 courses | GP | WHO | Unclear | ①② |
| Wang LC 2015 | 40/40 | 52/28 | 35-75 | 32/34/14 | JOE+GP | 20-30ml/d,  15 days/course,  2 courses | GP | WHO | WHO | ①②③④ |
| Wang TX 2014 | 41/41 | 60/22 | 43-80 | 46/36/0 | SQFZ+NP | 250ml/d,  14 days/course,  2 courses | NP | Unclear | WHO | ①②③④⑤⑥⑦ |
| Wang YB 2015 | 56/52 | 56/52 | 35-75 | 58/50/0 | CKS+PP | 15ml/d,  14 days/course,  2 courses | PP | RECIST | Unclear | ①②③ |
| Wang YR 2016 | 96/96 | 132/60 | 38-70 | 73/91/28 | SF+GP | 100ml/d,  15 days/course,  4 courses | GP | RECIST | WHO | ①②④⑤⑥⑦⑧⑨ |
| Wang YY 2013 | 39/39 | 55/23 | 45-77 | 41/22/15 | KLT+GP | 200ml/d,  10 days/course,  2 courses | GP | WHO | WHO | ①②④⑤⑥⑦ |
| Wang YZ 2013 | 44/44 | 52/36 | 51-73 | Unclear | KA+PP/NP/GP | Unclear,  14 days/course,  2 courses | PP/NP/GP | RECIST | WHO | ①②③ |
| Wang ZH 2018 | 47/47 | 58/36 | E: 59.41±6.12  C: 58.72±6.64 | 31/63/0 | KA+AP | 60ml/d,  21 days/course,  3 courses | AP | WHO | Unclear | ①②⑥ |
| Wen HQ 2014 | 45/45 | 64/26 | 61-81 | 23/64/3 | AD+GP | 50ml/d,  21 days/course,  2 courses | GP | RECIST | NCI-CTC | ①②③⑦ |
| Wu L 2004 | 30/30 | 46/14 | 32-80 | 32/25/3 | SQFZ+PP/PC/NP | 250ml/d,  21 days/course,  2-3 courses | PP/PC/NP | WHO | Unclear | ①② |
| Wu ZY 2019 | 28/28 | 29/27 | 38-71 | 17/25/14 | SQFZ+GP | 250ml/d,  21 days/course,  2 courses | GP | WHO | WHO | ①②③④⑤⑥ |
| Xiao XJ 2020 | 38/37 | 41/34 | 50-69 | Unclear | KLT+PC | 200ml/d,  21 days/course,  2 courses | PC | Unclear | Unclear | ①②⑦ |
| Xing HJ 2014 | 60/60 | 74/46 | 62-78 | Unclear | AD+NP | 50ml/d,  14 days/course,  2 courses | NP | WHO | WHO | ①②③④⑤⑥⑦ |
| Xiong XF 2021 | 48/48 | 49/47 | 60-81 | 14/65/17 | AD+PP | 50-100ml/d,  Unclear,  Unclear | PP | Unclear | Unclear | ①② |
| Xu DM 2021 | 55/50 | 59/46 | 45-74 | Unclear | AD+PP/GP | 400ml/d,  Unclear,  Unclear | PP/GP | Unclear | Unclear | ①②⑦ |
| Xu JL 2018 | 50/50 | 53/47 | 30-75 | 47/51/2 | LXX+GP | 400mg/d,  7 days/course,  3 courses | GP | Unclear | WHO | ①②⑤ |
| Xu L 2021 | 63/63 | 73/53 | 41-76 | 0/126/0 | SM+AC | 60ml/d,  7 days/course,  4 courses | AC | Unclear | Unclear | ①②⑦ |
| Xu Y 2017 | 37/37 | 44/30 | 57-83 | Unclear | CKS+GP | 20ml/d,  21 days/course,  2 courses | GP | RECIST | Unclear | ①②④⑥ |
| Xu ZJ 2020 | 40/40 | 53/27 | 49-72 | 23/57/0 | AD+GP | 50-100ml/d,  21 days/course,  2 courses | GP | Unclear | Unclear | ①②④⑥ |
| Yang B 2014 | 25/25 | 25/25 | 34-76 | 21/19/10 | CKS+NP | 20ml/d,  10 days/course,  2 courses | NP | WHO | Unclear | ①②④⑥⑦⑧ |
| Yang GW 2010 | 115/41 | 95/61 | E: 63.40±9.49  C: 60.98±8. 49 | 30/116/10 | KLT+NP/TC/DC | 200ml/d,  14 days/course,  2 courses | NP/PC/DC | WHO | Unclear | ①②③ |
| Yan QH 2018 | 49/49 | 63/35 | 38-76 | 47/51/0 | KLT+GP | 200ml/d,  14 days/course,  4 courses | GP | RECIST | Unclear | ①②④ |
| Yao DJ 2013 | 50/50 | 84/16 | 30-70 | 27/73/0 | SQFZ+GP | 250ml/d,  28 days/course,  1 courses | GP | WHO | Unclear | ①②③④⑤⑥ |
| Ye CY 2019 | 40/40 | 54/26 | 55-77 | 35/40/5 | KLT+GP | 200ml/d,  10 days/course,  2 courses | GP | RECIST | Unclear | ①②⑦ |
| Ye HN 2015 | 54/53 | 68/39 | E: 65.4±12.7 C: 64.7±11.8 | 34/52/21 | JOE+GP | 30ml/d,  14 days/course,  4 courses | GP | RECIST | WHO | ①②④⑧ |
| Ye ZK 2018 | 33/33 | 45/21 | E: 66. 36±3. 21  C: 66. 44±3. 36 | Unclear | KA+PP | 80-120ml/d,  30 days/course,  2-5 courses | PP | Unclear | Unclear | ①②④⑦ |
| Yu F 2007 | 30/30 | 44/16 | 50-78 | 31/19/10 | SQFZ+DP | 250ml/d,  10 days/course,  2-3 courses | DP | WHO | WHO | ①②④⑤⑥ |
| Yu FM 2018 | 40/40 | 51/29 | 43-74 | 53/27/0 | KA+DP | 40ml/d,  21 days/course,  2 courses | DP | WHO | Unclear | ①②④⑥⑦ |
| Zhang H 2009 | 41/41 | 53/29 | 30-76 | Unclear | AD+PP | 50ml/d,  Unclear,  2 courses | PP | Unclear | Unclear | ①②③ |
| Zhang HF 2019 | 77/77 | 83/71 | 45-82 | 77/77/0 | CKS+PP | 20ml/d,  14 days/course,  2 courses | PP | RECIST | NCI-CTC | ①②④⑤⑥⑦⑨ |
| Zhang J 2015 | 44/44 | 48/40 | 55-87 | 0/88/0 | AD+AP | 50ml/d,  14 days/course,  Unclear | AP | RECIST | WHO | ①②③④⑤⑥ |
| Zhang JJ 2015 | 42/42 | 48/36 | 28-78 | Unclear | CKS+PP | 20ml/d,  14 days/course,  3 courses | PP | RECIST | Unclear | ①② |
| Zhang JL 2010 | 30/30 | 45/15 | 51-78 | 37/15/8 | KA+PP | 40ml/d,  14 days/course,  2 courses | PP | WHO | WHO | ①②③⑦ |
| Zhang KS 2021 | 48/48 | 55/41 | 45-78 | 51/41/4 | KLT+GP | 200ml/d,  21 days/course,  Unclear | GP | RECIST | Unclear | ①②⑤⑥⑦ |
| Zhang MM 2019 | 50/50 | 52/48 | E: 61.25±6.35  C: 60.34±7. 02 | 100/0/0 | KLT+GP | Unclear,  20 days/course,  Unclear | GP | RECIST | Unclear | ①②④⑤⑦ |
| Zhang MY 2019 | 52/48 | 53/47 | 51-76 | 36/60/ 4 | CKS+GP | 20ml/d,  14 days/course,  2 courses | GP | Unclear | Unclear | ①②③④ |
| Zhang SC 2021 | 45/45 | 59/31 | 60-79 | 23/47/20 | KA+PP/GP | 60ml/d,  14 days/course,  2 courses | PP/GP | Unclear | Unclear | ①②③ |
| Zhang ZJ 2019 | 41/41 | 48/34 | 31-59 | 0/61/21 | CKS+AP | 12ml/d,  21 days/course,  4 courses | AP | RECIST | Unclear | ①② |
| Zhao J 2019 | 43/43 | 55/31 | E: 64.02±2.34  C: 63.26±2.57 | 43/39/4 | AD+GP | 50mg/d,  21 days/course,  2 courses | GP | UICC | Unclear | ①② |
| Zhao K 2012 | 43/43 | 61/25 | 50-78 | 25/51/10 | CKS+GP | 12ml/d,  14 days/course,  2 courses | GP | Unclear | Unclear | ①② |
| Zhao LL 2018 | 41/41 | 49/33 | 41-69 | 34/25/23 | SM+GP | 100mg/d,  10 days/course,  3 courses | GP | RECIST | NCI-CTC | ①②⑥⑦ |
| Zhao Q 2019 | 52/52 | 59/45 | E: 64.2±6.4  C: 64.5±6.2 | 37/62/5 | SQFZ+PP | 250ml/d,  21 days/course,  2 courses | PP | WHO | Unclear | ①②③④⑦ |
| Zhao WY 2013 | 35/36 | 50/21 | 26-74 | 23/43/5 | LE+GP | 1mg/d,  6 days/course,  2 courses | GP | RECIST | WHO | ①②③ |
| Zhao ZY 2014 | 50/52 | 80/22 | E: 57.3±8.1  C: 59.9±7.4 | 65/37/0 | SQFZ+GP | 250ml/d,  10-14 days/course,  2-6 courses | GP | RECIST | WHO | ①②③④⑤⑥⑦ |
| Zhou YZ 2020 | 40/40 | 42/38 | E: 55.2± 9.6  C: 55.4±8.7 | 0/80/0 | LXX | 600mg/d,  7 days/course,  4 courses | AP | RECIST | WHO | ①②④⑥⑦ |
| Zhu JJ 2016 | 43/42 | 49/36 | 48-80 | 38/45/2 | KLT+GP | 200ml/d,  10 days/course,  2 courses | GP | WHO | NCI-CTC | ①②④⑤⑥⑦ |
| Zhu JL 2017 | 84/84 | 95/73 | 31-75 | Unclear | AD+DC | 50ml/d,  14 days/course,  2 courses | DC | WHO | Unclear | ①②④⑥ |
| Zhu LM 2011 | 92/90 | 104/78 | 42-75 | 94/71/17 | CKS+PP/NP/GP | 20ml/d,  28 days/course,  3 courses | PP/NP/GP | WHO | WHO | ①②④⑤⑥⑧⑨ |
| Zhu QS 2006 | 30/30 | 32/28 | 33-74 | 25/33/2 | AD+DP | 50ml/d,  10 days/course,  3 courses | DP | RECIST | WHO | ①②④⑤⑥⑦⑧ |
| Zhu SC 2020 | 42/42 | 60/24 | E: 57.76±8.89  C: 57.40±7.96 | 0/84/0 | KA+AP | 40ml/d,  21 days/course,  4 courses | AP | RECIST | WHO | ①②⑦ |

NSCLC: non-small cell lung cancer; E/C: experimental group/control group; M/F: male/female; Sq/Ad/Ot: squamous carcinoma/adenocarcinoma/other; D/C: dose/cycles; RECIST: response evaluation criteria in solid tumors; NCI-CTC: National Cancer Institute Common Toxicity Criteria; WHO: World Health Organization; CTCAE: Common Terminology Criteria for Adverse Events; UICC: Union for International Cancer Control; AD: Aidi injection; CKS: Compound Kushen injection; HCS: Huachansu injection; JOE: Javanica oil emulsion injection; KLT: Kanglaite injection; KA: Kangai injection; LXX: Lanxiangxi injection; LE: Lentinan injection; SF: Shenfu injection; SM: Shenmai injection; SQFZ: Shenqifuzheng injection; XAP: Xiaoaiping injection; ①: ORR, objective response rate; ②: DCR, disease control rate; ③: QoL, quality of life; ④: leukopenia; ⑤: anemia; ⑥: thrombocytopenia; ⑦: nausea and vomiting; ⑧: diarrhea; ⑨: constipation;
